# Antioxidant-related enzymes and peptides as biomarkers of metallic nanoparticles (eco)toxicity in the aquatic environment

**DOI:** 10.1016/j.chemosphere.2024.142988

**Published:** 2024-09

**Authors:** Tomas Do, Silvia Vaculciakova, Katarzyna Kluska, Manuel David Peris-Díaz, Jan Priborsky, Roman Guran, Artur Krężel, Vojtech Adam, Ondrej Zitka

**Affiliations:** a Department of Chemistry and Biochemistry, Faculty of AgriSciences, Mendel University in Brno, Zemedelska 1, 613 00, Brno, Czech Republic; b Department of Chemical Biology, Faculty of Biotechnology, University of Wrocław, Joliot-Curie 14a, 50-383, Wrocław, Poland

**Keywords:** Oxidative stress, Glutathione, Metallothionein, Mass spectrometry, Aquatic organism

## Abstract

Increased awareness of the impact of human activities on the environment has emerged in recent decades. One significant global environmental and human health issue is the development of materials that could potentially have negative effects. These materials can accumulate in the environment, infiltrate organisms, and move up the food chain, causing toxic effects at various levels. Therefore, it is crucial to assess materials comprising nano-scale particles due to the rapid expansion of nanotechnology. The aquatic environment, particularly vulnerable to waste pollution, demands attention. This review provides an overview of the behavior and fate of metallic nanoparticles (NPs) in the aquatic environment. It focuses on recent studies investigating the toxicity of different metallic NPs on aquatic organisms, with a specific emphasis on thiol-biomarkers of oxidative stress such as glutathione, thiol- and related-enzymes, and metallothionein. Additionally, the selection of suitable measurement methods for monitoring thiol-biomarkers in NPs' ecotoxicity assessments is discussed. The review also describes the analytical techniques employed for determining levels of oxidative stress biomarkers.

## Highlights

• Stress markers to monitor the toxicity of metallic nanoparticles.
• The behavior and fate of metallic nanoparticles in the aquatic environment.
• The analytical techniques used for monitoring levels of stress biomarkers.
• Metallomics as fundamental aspect of understanding the toxicity of a metal element.
• Standalone or hyphenated-MS techniques for analyzing metallothionein or metal ions.

## Graphical abstract

**Figure:**
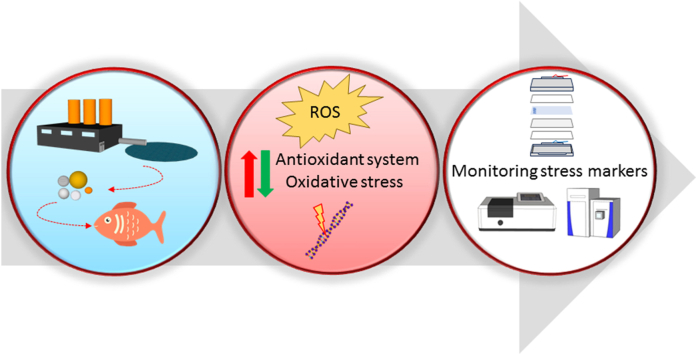

## 1 Introduction

**Fig. 1:**
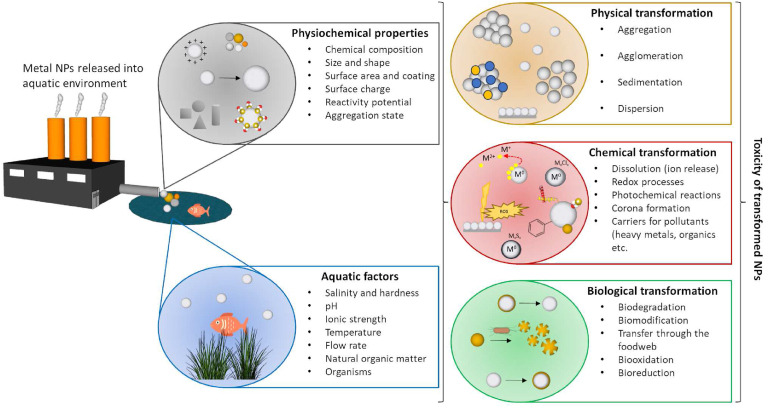
Summary of factors influencing the toxicity of nanoparticles (NPs) in the aquatic environment.

In recent years, inorganic nanoparticles (NPs) have demonstrated promising potential in a wide range of applications, including food storage containers (Abdulagatov et al., 2024; Zhou et al., 2024a), water remediation (Ajith et al., 2021), electronic devices (Du et al., 2018), cosmetics (Du et al., 2018; Ginzburg et al., 2018; Zhang et al., 2018a), textiles (Yetisen et al., 2016), imaging (Shen et al., 2024; Xiang et al., 2024), and drug delivery (Patra et al., 2018; Yang and Chen, 2022; Chu et al., 2024). NPs, characterized by dimensions ranging from 1 to 100 nm, exhibit exceptional physical and chemical properties that are not solely dependent on the composition of their constituent atoms but also on their modifiable surface characteristics. However, the escalating use of NPs brings forth potential environmental risks as they enter our ecosystem. Consequently, a comprehensive assessment of the physicochemical properties and overall impact of NPs becomes imperative. Despite the increasing number of studies addressing NPs' toxicity, a clear consensus regarding their exact mechanisms of toxicity has yet to be established. Factors such as concentration (Farkas et al., 2011; Khan et al., 2017a; Tang et al., 2019; Sibiya et al., 2022a), size (Zhang et al., 2022b; Windell et al., 2023; Lira et al., 2024), shape (George et al., 2012; Afrooz et al., 2013; Toy et al., 2014; Auffan et al., 2020; Lira et al., 2024), surface coating (Sharma et al., 2014; Hou et al., 2017; Barreto et al., 2019; Windell et al., 2023), biostability, the ability to release free metal ions (Djurisic et al., 2015; Khoshnamvand et al., 2020; Lee et al., 2021; Yang et al., 2022), photochemical properties (Clemente et al., 2015; Joo and Zhao, 2017; Horie and Tabei, 2021), and sorption properties (e.g., Trojan horse effect) (Monopoli et al., 2012; Ding et al., 2018; Naasz et al., 2018; Stepien et al., 2018; Wheeler et al., 2021) all contribute to the toxicity of NPs (Fig. 1).

As mentioned earlier, nanotechnology is a rapidly expanding market segment, leading to an increased presence of nanosized particles in the environment (Zhu et al., 2019). Among the most critical areas of concern are the potential effects of materials in aquatic environments, which often bear the burden of waste and may introduce various unwanted substances into the human body. Notably, NPs possess dispersibility in water, which could adversely impact the aquatic environment. Consequently, investigating nanoparticle toxicity in aquatic environments has become a topic of significant interest in the field of ecotoxicology. Silver (Ag) (Farkas et al., 2011; Sharma et al., 2015; Banumathi et al., 2017; Khan et al., 2017a, 2018; Lacave et al., 2017; Pecoraro et al., 2017; Valerio-Garcia et al., 2017; Renuka et al., 2020; Babaei et al., 2022; Kokturk et al., 2022; Lu et al., 2022; Sibiya et al., 2022a; Sekar et al., 2023; Kakakhel et al., 2024), copper oxide (CuO) (Lu et al., 2017; Noureen et al., 2018; Johari et al., 2020; Zhao et al., 2020) and titanium dioxide (TiO_2_) (Gondikas et al., 2014; Canli et al., 2018; Huang et al., 2018b; Delmond et al., 2019; Chen et al., 2019; Saidani et al., 2019; Souza et al., 2019; Tang et al., 2019; Roy et al., 2020; Dellali et al., 2021) are examples of extensively studied NPs that pose potential threats to the aquatic environment. To assess the toxicological effects of NPs, several biomarkers of oxidative stress have been explored, with particular attention given to thiol-based biomarkers such as glutathione, thiol- and related-enzymes, and metallothionein (Sharma et al., 2015; Banumathi et al., 2017; Khan et al., 2017a, 2018; Lacave et al., 2017; Pecoraro et al., 2017; Valerio-Garcia et al., 2017; Sekar et al., 2023; Kakakhel et al., 2024). Furthermore, the selection of suitable measurement methods for monitoring thiol-biomarkers in NPs' ecotoxicity assessments is a critical aspect of research in this field. Additionally, the review describes the analytical techniques employed for determining levels of oxidative stress biomarkers.

### 1.1 Methodological approaches in the assessment of NPs and their effects

The comprehensive characterization of synthesized NPs and a deeper understanding of their nature, fate, and environmental impact necessitate the adoption of multidisciplinary analytical approaches. Various analytical methods are suitable for NPs' studies. For determining the physical form and morphology of NPs, transmission electron microscopy (TEM) (Seo and Park, 2018), dynamic light scattering (DLS) (Turley et al., 2018), scanning electron microscopy (SEM) (Odziomek et al., 2017), and atomic force microscopy (AFM) (Christensen et al., 2015) are commonly used. Alternatives to DLS include NPs tracking analysis (NTA) (Nguyen and Bon, 2024), photon correlation spectroscopy (PCS) (Urnukhsaikhan et al., 2021), differential centrifugal sedimentation (DCS), and multi-angle light scattering (MALS) (Klein et al., 2020). Structural analysis employs methods as X-ray powder diffraction (XRD), Fourier-transform infrared spectroscopy (FTIR) (Saratale et al., 2018), Raman spectroscopy (Johnston et al., 2010; Testa-Anta et al., 2019; Tokoro et al., 2022), small angle X-ray scattering (SAXS) (Li et al., 2016b) and nuclear magnetic resonance (NMR) (Kunc et al., 2019). Surface analysis can be supported by Auger electron spectroscopy (AES) and Brunauer-Emmett-Teller (BET) analysis (Rades et al., 2014). Additionally, separation techniques like as polyacrylamide gel electrophoresis (PAGE) or agarose gel electrophoresis (AGE), density gradient centrifugation (DGC), field flow fractionation (FFF), gel permeation chromatography (GPC), size exclusion chromatography (SEC) and hydrodynamic chromatography (HDC) are used to isolate and analyze NPs (Philippe and Schaumann, 2014; Mori, 2015; Meisterjahn et al., 2016). Optical properties of NPs are typically assessed using UV–Vis spectroscopy (Begum et al., 2018) and photoluminescence spectroscopy (Xue et al., 2014). To determine chemical composition and electronic states, X-ray photoelectron spectroscopy (XPS) (Nguyen et al., 2018), energy-dispersive X-ray spectroscopy (EDX), and wavelength dispersive X-ray spectroscopy (WDX) are commonly utilized (Slater et al., 2016). Furthermore, fluorescence methods provide valuable information about the size, shape, surface properties, and interactions of NPs with other molecules. Fluorescence techniques used in nanoparticle characterization include fluorescence spectroscopy, confocal microscopy, fluorescence correlation spectroscopy (FCS), and Förster resonance energy transfer (FRET) (Kaeokhamloed et al., 2022; Schmitt et al., 2022; Gupta et al., 2024). Moreover, mass spectrometry (MS) techniques serve as powerful tools for characterizing and determining the composition and structure of NPs, owing to their ability to measure various sample types, high sensitivity, mass resolution, and compatibility with front-end separation techniques. The importance of utilizing MS techniques in NPs' characterization continues to grow. For example, ion-mobility mass spectrometry (IM-MS) analyzes particle size and charge (Kapellios and Pergantis, 2012). Inductively coupled plasma mass spectrometry (ICP-MS) and inductively coupled optical emissions spectrometry (ICP-OES) provides detailed information on nanoparticle concentration, size distribution, and elemental composition. In its single particle ICP-MS (spICP-MS) configuration, ICP-MS can detect and precisely quantify NPs at the individual particle level (Degueldre and Favarger, 2003; Mitrano et al., 2012; Borovinskaya et al., 2014; Peters et al., 2014; Tharaud et al., 2017; Merrifield et al., 2018). Furthermore, nanoscale secondary ion mass spectrometry (NanoSIMS) along with EDX and X-ray fluorescence microscopy (XRF) are effective methods for elemental mapping inside cells and intracellular compartments (Deniaud, 2021). Other MS methods offer additional insights into the interactions, transformations, and biological effects of NPs. Together, these techniques provide a comprehensive toolkit for studying NPs and their impacts in diverse fields of research and application. In the last two decades, computational modeling methods have been developed for nanoparticle evaluation and measurement in the environment (Blaser et al., 2008; Mueller and Nowack, 2008; Koelmans et al., 2009; Johnson et al., 2011; Musee, 2011; Arvidsson et al., 2012), with probabilistic approaches being particularly valuable (Gottschalk et al., 2010a, 2010b; Sun et al., 2017). This technique involves tracking the time-dependent flow of specific NPs through technical modules and environmental compartments, allowing the detection of several nanograms to micrograms of NPs in water samples.

### 1.2 General biochemistry of NPs

**Fig. 2:**
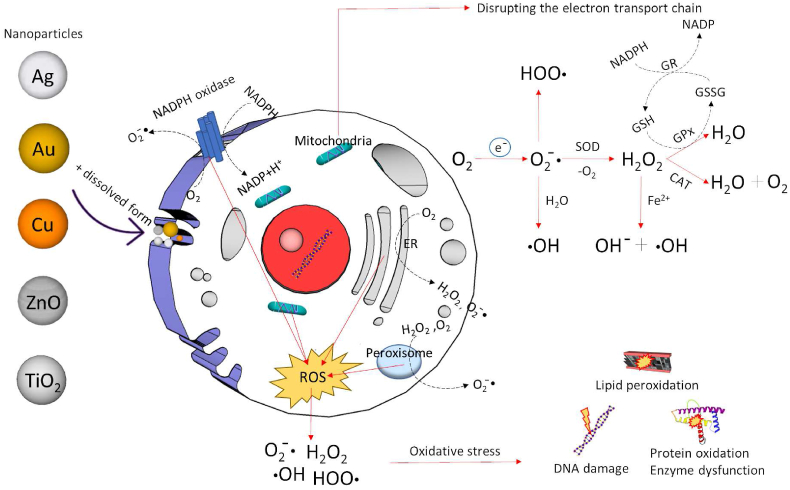
Possible routes of NPs' entry into eukaryotic cells: uptake, trafficking, and stress generation. NPs interact with the plasma membrane, peroxisomes, endoplasmic reticulum (ER), and mitochondria, leading to the generation of reactive oxygen species (ROS). ROS can cause severe damage to proteins, lipids, enzymes, and DNA, ultimately triggering an inflammatory process and cell death. The negative toxic effects of ROS can be counteracted by various antioxidant enzymes. Superoxide dismutase (SOD) catalyzes the conversion of O_2_^−^• to H_2_O_2_. H_2_O_2_, one of the main ROS, is further converted to H_2_O and O_2_ by catalase (CAT) or glutathione peroxidase (GPx). GPX and glutathione reductase (GR) are essential for glutathione (GSH) metabolism. In the presence of metal ions, •OH is formed via the Fenton reaction.

The induction of oxidative stress has been a focal point in the study of NPs' toxicity (Khan et al., 2017b; Liu et al., 2017; Lu et al., 2017; Valerio-Garcia et al., 2017; Huang et al., 2018b; Pitt et al., 2018; Delmond et al., 2019; Saidani et al., 2019; Tang et al., 2019; Renuka et al., 2020; Sibiya et al., 2022a; Sekar et al., 2023). Oxidative stress occurs when there is an imbalance or disruption in the control of reactive oxygen species (ROS), resulting in the accumulation of free radicals and non-radical forms of ROS. In eukaryotic organisms, most oxygen is reduced to water in the electron transport chain through a four-electron reduction process. However, some oxygen molecules can be transformed via one-electron pathways into superoxide anion (O_2_^−^•), hydrogen peroxide (H_2_O_2_), and hydroxyl radical (•OH), collectively known as reactive oxygen species (ROS). ROS also include singlet oxygen (^1^O_2_), hydroperoxide radical (HO_2_^−^•), and peroxyl radical (ROO•) (Lushchak, 2014b). Oxidative stress is defined as a state where there is an acute or chronic increase in ROS concentration, leading to oxidative modification of cellular components and disruption of cellular metabolism and regulatory pathways (Lushchak, 2016). ROS can originate from various sources, including endogenous and exogenous factors. Endogenous sources include mitochondria (Kroller-Schon et al., 2014), peroxisomes, microsomes (Buonocore et al., 2010), enzyme systems such as NADPH oxidase (Kroller-Schon et al., 2014), xanthine oxidase, and nitric oxide synthase (Buonocore et al., 2010). Inflammatory processes involving neutrophils, eosinophils, and macrophages also contribute to ROS production (de Groot et al., 2019). Exogenous factors include atmospheric pollutants, UV radiation, gamma radiation, and X-ray radiation (Valko et al., 2006). NPs can induce ROS generation through three main mechanisms. The first mechanism involves the reactivity of released metal ions on the NPs' surface, leading to ROS generation via the Fenton reaction, which involves a multi-step reaction between H_2_O_2_ and metal ions, particularly divalent metals (M^II^), resulting in the formation of hydroxyl radicals (Valko et al., 2006; Bokare and Choi, 2014; Cao et al., 2021). The second mechanism entails NPs interacting with cellular targets and accumulating in specific locations, such as direct interaction with plasma membrane NADPH oxidase or mitochondria, disrupting the electron transport chain (Lushchak, 2014b). The third mechanism involves an inflammatory response, where ROS is initially generated through one of the aforementioned mechanisms, leading to inflammation and the release of additional ROS from inflammatory cells (Yu et al., 2020). Oxidative stress exerts harmful effects on living organisms. Depending on the level of oxidative stress, it can result in DNA damage, lipid peroxidation, activation of cell signaling pathways, decreased cell growth, tissue fibrosis, and the development of cancer (Lushchak, 2014a) (Fig. 2).

To protect against oxidative stress, organisms induce the production of ROS through scavenging mechanisms involving various antioxidants. Antioxidants can be categorized into two groups based on their molecular weight. The first group consists of small molecular mass antioxidants, including vitamins A, C, and E. Another crucial antioxidant is reduced glutathione (GSH). The second group comprises larger molecules such as superoxide dismutase (SOD), catalase (CAT), glutathione peroxidase (GPx), glutathione reductase (GR), and glutathione transferase (GST). The concentration of antioxidants reflects the cellular antioxidant defense status and stability against oxidative stress. Thus, the expression and activity of these enzymes serve as indicators for evaluating oxidative stress induction (Lushchak, 2014b, 2016). In vitro studies on various cell cultures (including human and other mammals) have consistently demonstrated cytotoxicity and ROS production in response to NPs, such as Ag (Liu et al., 2010; Zhang et al., 2016; Akter et al., 2018), TiO_2_ (Alinovi et al., 2015; Thai et al., 2019), zinc oxide (ZnO) (Hong et al., 2013; Pandurangan and Kim, 2015; Pinho et al., 2020), and cerium oxide (CeO_2_) (Demokritou et al., 2013; Ngoc et al., 2019; Thai et al., 2019) in human bronchial cell lines 16HBE, human lung epithelial cells, fibroblasts, spermatogonia cells, NIH3T3, alveolar epithelial cells of the SV40T2 line, alveolar macrophages, renal epithelial cells, and PK-LUC. In vivo experiments have also demonstrated ROS generation following exposure to NPs (Delmond et al., 2019; Zhao et al., 2020; Hong et al., 2022; Lu et al., 2022). AgNPs have also been found to impact endocrine functions (Ferdous and Nemmar, 2020; Deniaud, 2021). Aquatic organisms have also been widely employed as animal models for NPs' toxicity investigations. Zebrafish (*Danio rerio*) has been the most commonly used fish model (Lacave et al., 2017; Pecoraro et al., 2017; Tang et al., 2019; Renuka et al., 2020; Zhao et al., 2020; Kokturk et al., 2022), while *Daphnia magna* has been established as a well-suited aquatic model for NPs' toxicity studies (Lu et al., 2017; Chen et al., 2019; Liu et al., 2022). Carp (*Cyprinus carpio*) and other aquatic organisms such as *Oncorhynchus mykiss* (Farkas et al., 2011; Chupani et al., 2018; Noureen et al., 2018; Razmara et al., 2023; Sekar et al., 2023; Kakakhel et al., 2024), *Labeo Rohita* (Khan et al., 2017a; Aziz and Abdullah, 2023), *Chapalichthys pardalis* (Valerio-Garcia et al., 2017), *Ruditapes decussatus* (Fkiri et al., 2018; Saidani et al., 2019), *Mytilus coruscus* (Huang et al., 2018b), *Astyanax serratus* (Delmond et al., 2019), Nile Tilapia (*Oreochromis niloticus*) (Abdelazim et al., 2018), and *Carassius auratus* (Benavides et al., 2016) have also been utilized. It is worth noting that the assessment of NPs' toxicity is a complex matter that depends not only on the structural properties of NPs but also on the design of toxicity testing. Therefore, it is crucial to evaluate results for each type of NPs separately and avoid generalizations. This review article focuses on the effects of different NPs on oxidative stress, with an emphasis on the classification of biomarkers used to evaluate oxidative stress in aquatic organisms.

## 2 Metallic nanoparticles in the aquatic environment

**Fig. 3:**
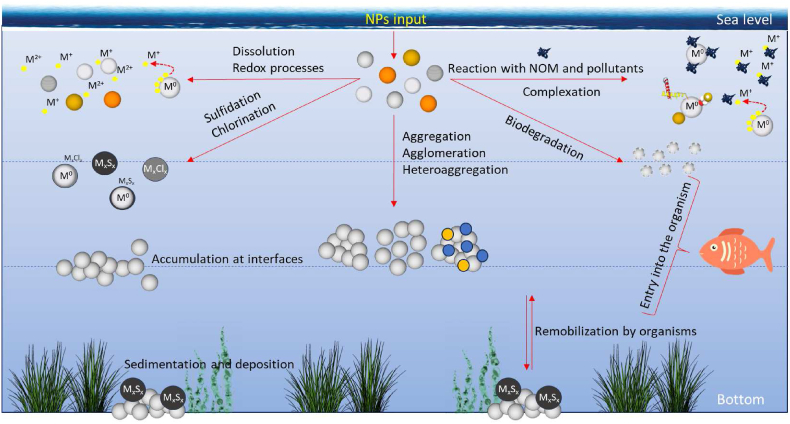
The fate of NPs in the aquatic environment. Several possible transformations occur when NPs are released into the aquatic environment. In addition to aggregation and agglomeration, their dissolution and subsequent formation into ions and salts are also observed in the aquatic ecosystem. NPs can be bioavailable to aquatic organisms, plants, and/or adsorbed on the surface of water bottoms and/or accumulated at water interfaces. The chemical species of the given element and the properties of the environment determine the bioavailability and toxicity of NPs (inspired by (Klaine et al., 2008)).

The relationship between the behavior of metal NPs and their toxic effects in aquatic environments is a crucial area of research in nanotoxicology. Once NPs enter the aquatic environment, the particles are affected by transformations and transport mechanisms, which can be divided into three major groups: i) physical transformation including aggregation, agglomeration, sedimentation, dispersion, ii) chemical transformation including dissolution, adsorption on the surface of natural NPs, redox processes, photochemical reactions, corona formation, and iii) biological transformations including biodegradation, or biomodification (Fig. 3) (Klaine et al., 2008; Devasena et al., 2022).

The behavior of a given type of NPs in the environment depends on factors such as its physicochemical properties, aggregation ability, route and time of exposure, dose-response, or features of the environment in which NPs are present. Particle size and surface area are key properties that influence the fate and transport of metal NPs in the environment. The small size of NPs typically prevents them from settling due to gravity, allowing them to stay suspended in aquatic environment. Additionally, particle size has been shown to influence the endocytic pathway, cellular uptake, and particle efficiency (Turan et al., 2019). As particle size decreases, the surface-to-volume ratios tends to increase. This larger surface area benefits NPs by enhancing their reactivity, which has impact on their fate and transport. Small-sized particles are frequently reported to cause more oxidative stress and greater DNA damaging compared to larger particles (Pan et al., 2009; Ahn et al., 2014; Angelstorf et al., 2014; Kim et al., 2014; Santo et al., 2014; Arratia et al., 2019). However, even if particles are within the nanoscale range, those that are physically and chemically inactive do not cause oxidative stress. Therefore, particle size alone is not a direct factor in inducing oxidative stress from NPs (Horie and Tabei, 2021). Particle shape also has been reported as factor that affects NPs' toxicity (Simon-Deckers et al., 2009; George et al., 2012; Cui et al., 2017). For example, octahedral Cu_2_O nanocrystal showed higher oxidative stress than cubic (Fan et al., 2012). In the aquatic environment, there are factors such as salinity, pH, ionic strength, temperature, hardness, flow rate, dissolved gases, presence/absence of living organisms, or other components of the aqueous medium, which affect the transport and subsequent toxicity of metallic NPs (Fig. 3). Thus, there is a difference between the NPs' toxicity in seawaters and freshwaters. Ionic strength is significantly higher in seawaters than in freshwaters. Therefore, in freshwater systems with low ionic strength, NPs may remain more dispersed and mobile. In contrast, in marine environments with high ionic strength, NPs are more likely to aggregate (Diegoli et al., 2008; Lapresta-Fernandez et al., 2012; Ghobashy et al., 2021; Wang and Liu, 2022). Aggregation refers to the process where NPs clump together to form larger particles or aggregates. According to B. Derjaguin, L. Landau, E. Verwey, and T. Overbeek (DLVO) theory, particles are held in suspension by a balance of attractive and repulsive forces. DLVO explains the aggregation of an aqueous dispersion quantitatively and describes the net interaction energy between charged surfaces interacting with a liquid medium. It combines the effects of van der Waals attraction and electrostatic repulsion due to the so-called electrostatic double-layer force (Nel et al., 2009). The degree of aggregation is a critical factor that affects the overall toxicity of NPs. During the aggregation/agglomeration process, the surface area of the individual particles and the interfacial free energy are reduced, which can reduce their reactivity and interaction with biological systems. (Lapresta-Fernandez et al., 2012; Spurgeon et al., 2020). As individual NPs have high surface reactivity, they can induce greater oxidative stress by generating ROS. Aggregated NPs, with their reduced surface area, might exhibit lower ROS generation, potentially decreasing their toxicity (Lowry et al., 2012). Moreover, as agglomerates and aggregates tend to sediment, this property must also be monitored and controlled. For example, Auffan et al. tested the toxicity of CeO_2_ NPs to *Daphnia pulex*. The authors found that the actual exposure time in the water was only 2 h because NPs were removed from the water column by rapid agglomeration and sedimentation (Auffan et al., 2013). However, this sedimentation can have adverse effects on benthic organisms, which live in or on the sediment. These organisms may ingest the aggregated NPs, leading to physical blockages or toxic effects from the particles themselves or from dissolved ions released from the NPs (Wang and Liu, 2022). Bioavailability of NPs is another aspect influenced by aggregation. Dispersed NPs are more bioavailable and can be readily taken up by aquatic organisms through various exposure routes, including ingestion, inhalation, and dermal absorption. Aggregated NPs may have reduced bioavailability but can still be taken up by filter-feeding organisms or through the food chain. This can lead to bioaccumulation and biomagnification, where NPs and their toxic effects become more concentrated as they move up the food chain, potentially impacting higher trophic levels, including humans (Ward and Kach, 2009; Croteau et al., 2014; Babaei et al., 2022; Sibiya et al., 2022b; Vineeth Kumar et al., 2022). Overall, while aggregation generally reduces the immediate reactivity and bioavailability of NPs, it does not eliminate their potential for causing harm. Conversely, when agglomerates or aggregates enter or form within biological systems, they appear to exhibit high toxic potential. In the study by *Zhu* et al., it was found that *Danio rerio* embryos exposed to agglomerates of Fe_3_O_4_ NPs showed higher mortality and a higher incidence of morphological malformations compared to stabilized particles (Zhu et al., 2012). Moreover, NPs can also aggregate with other particles, such as natural organic matter (NOM), or other pollutants, rather than with themselves. This important process called heteroaggregation significantly influences the behavior, distribution, and toxicity of NPs (Batley et al., 2013). The environmental water chemistry also significantly impacts the redox reactions which subsequently influence dissolution and complexation with other chemical constituents by changing the oxidation state of metal NPs, thereby affecting their reactivity. The dissolution of the material is strongly affected by the pH, the ionic strength of the medium, and the presence of inorganic and organic compounds (Lowry et al., 2012; Lee et al., 2018; Mbanga et al., 2022; Yadav et al., 2022). For example, ions released from the surface of NPs such as NPs of Ag, ZnO, or CuO often contribute greatly to the toxicity of the material leading to oxidative stress, membrane damage, enzyme inhibiton and DNA damage (Liu and Hurt, 2010b; Wang et al., 2016a, 2021b; Xiao et al., 2016; Kalantzi et al., 2019; Yang et al., 2022). Nevertheless, the presence of NOM can react with the dissolved ions, influencing their bioavailability and toxicity. One of the major components of NOM, humic acid (HA), can adsorb onto the surface of NPs, leading to changes in their surface properties (Baalousha, 2009). This adsorption can stabilize NPs, preventing aggregation and increasing their dispersion in aquatic environments (Diegoli et al., 2008; Chappell et al., 2009; Zhang et al., 2009; Loosli et al., 2013; Wang and Liu, 2022). Additionally, HA can affect the redox state of NPs, altering their oxidative potential and ability to generate ROS. Research has shown that the presence of HA can both mitigate and enhance the toxicity of various types of NPs. For instance, it can form complexes with Ag^+^ ions released from NPs or reduce Ag^+^ to AgNPs, potentially reducing their free ion concentration and thereby modulating their toxicity (Liu and Hurt, 2010a; Akaighe et al., 2011; Gao et al., 2012; Hou et al., 2013; Yin et al., 2014; Cáceres-Vélez et al., 2018; Liu et al., 2018; Dong et al., 2020). Conversely, other studies have found that HA can increase the toxicity of ZnO and TiO_2_ NPs leading to increased oxidative stress in exposed aquatic organisms (Yang et al., 2013; Seitz et al., 2015; Akhil and Sudheer Khan, 2017). Some studies reported increased aggregation rate in presence HA and other pollutants (Wang et al., 2019a). The stability of NPs once dispersed in solutions is positively affected by their surface modifications (surfactants, polymers, polyelectrolytes, etc.), and this also significantly affects the interactions of particles with natural colloids (Kittler et al., 2010; Sharma et al., 2014). In addition to dissolution, sulfidation is one of the important and well-described abiotic transformation processes. Sulfidation depends on redox conditions, and Ag, ZnO, CuO, and Fe_3_O_4_ NPs are most prone to sulfidation, which occurs most often in the biological stage of wastewater treatment (WWTP), reducing the solubility, mobility, and thus the bioavailability of NPs (Ma et al., 2014; Baun et al., 2017; Lee et al., 2021; Zhang et al., 2022a). Therefore, sulfidation can significantly reduce the ROS-generating potential of NPs (Ma et al., 2013; Lee et al., 2021). For example, studies have shown that Ag_2_S are significantly less toxic to fish and invertebrates than their unsulfidized forms due to reduced ROS production (Devi et al., 2015). However, sulfidized NPs are more stable, which means they can remain in the environment for longer periods. NPs undergo other chemical transformations including phosphatisation, carbonation, chlorination, that significantly influence their behavior and toxicity. All these transformations play a crucial role in determining the effect of NPs on aquatic organism.

### 2.1 Titanium dioxide nanoparticles

TiO_2_ NPs have been extensively studied for their toxicity in the aquatic environment. Natural forms of TiO_2_ include rutile, anatase, and brookite, and the reported concentrations of titanium in rivers and seawater typically range between 0.01 and 5.5 μg/L (Monteiro et al., 2019). This low concentration is attributed to the low solubility of TiO_2_ in water. However, the increased use of TiO_2_ NPs in products such as sunscreens (Gondikas et al., 2014) and their application in solar devices as photocatalysts (Lan et al., 2013) are expected to result in higher TiO_2_ concentrations in the aquatic environment. For instance, when sunscreen is applied and comes into contact with water, TiO_2_ NPs can be washed into the aqueous environment. In a study by Gondikas et al., the concentration of TiO_2_ NPs released from sunscreen products into the Old Danube Lake (Vienna, Austria) was evaluated over a one-year period. The analysis of collected suspended particles using various techniques could not distinguish TiO_2_ NPs from sunscreen products from naturally occurring titanium NPs. However, a slight overall increase in titanium particles was observed during the summer season (Gondikas et al., 2014). TiO_2_ NPs are also added to paints and pigments to enhance whiteness and provide additional properties such as chemical degradation and self-cleaning abilities. Environmental contamination can occur through traditional waste management processes such as sewage, waste incineration, and landfill discharge, potentially leading to the uptake of NPs by plants and marine organisms, which may be consumed by humans and animals.

**Fig. 4:**
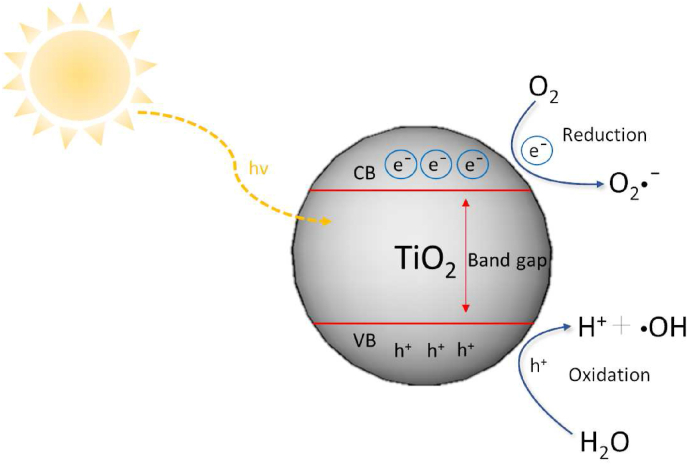
Schematic representation of the photochemical activation of TiO_2_ NPs and the generation of ROS potentially inducing oxidative stress. The light provides sufficient energy to TiO_2_ NPs that is equal to its band gap. The electron becoming excited, and these excited electrons move to the conduction band (CB), leaving behind positive charged holes (h^+^) in the valence band (VB). These species react with water or oxygen molecules to produce reactive oxygen species such as superoxide anion radicals (O_2_^−^•) and hydroxyl radicals (•OH).

Studies have demonstrated the accumulation of TiO_2_ NPs in aquatic organisms. For example, *Daphnia magna* exposed to 30 nm rutile TiO_2_ NPs at doses of 0.1, 1.0, and 10 mg/L showed higher bioconcentration factors (BCFs) at lower concentrations (0.1 and 1 mg/L) compared to higher concentrations (Fan et al., 2016). In another study, higher BCFs were observed in *Daphnia magna* exposed to anatase-rutile mixture (80:20) at a concentration of 1 mg/L compared to a concentration of 0.1 mg/L (Zhu et al., 2010). The presence of TiO_2_ NPs has also been shown to increase the accumulation of cadmium and arsenic in carp due to the strong sorption capacity of these elements (Dwivedi et al., 2015). Genotoxic effects were observed in the model organism *Danio rerio* after 14 and 21 days of exposure to TiO_2_ NPs at a concentration of 10 μg/L (Rocco et al., 2015). Long-term effects include the adhesion of TiO_2_-aggregates to the exoskeleton of *Daphnia magna*, which reduces their mobility and induces behavioral changes such as cyclic movements or hitting the vessel walls (Lovern et al., 2007). Changes in visual behavior patterns such as slowed speed and delayed response to stimuli was observed in zebrafish exposed to TiO2 NPs combined with bis(2-ethylhexyl)-2,3,4,5-tetrabromophthalate (Zhou et al., 2024b). Recent studies have investigated the impact of particle size and crystal forms of TiO_2_ NPs on their toxicity. The reported median lethal concentrations (LC_50_) for TiO_2_ NPs vary significantly depending on their size and crystal form. LC_50_ values for TiO_2_ NPs in *Daphnia magna* range from 29.8 μg/L to more than 250 mg/L within 48 h (Ma et al., 2012; Li et al., 2014a, 2016a; Wormington et al., 2017). In a study by Clément et al., the toxicity of TiO_2_ NPs of different sizes and crystal forms to *Daphnia magna* followed the order: 44 μm anatase <1 μm rutile <32 nm anatase <25 nm anatase <21 nm anatase-rutile mixture (80:20) < 15 nm anatase (Clement et al., 2013). Chen et al. observed that acute toxicity decreased with increasing rutile content, and both acute toxicity and BCFs decreased with increasing particle size (Chen et al., 2019). Furthermore, TiO_2_ NPs are known for their photocatalytic properties and for the photochemical induction of ROS when these NPs are exposed to light, especially UV radiation. Upon photoactivation, TiO_2_ NPs generate photoexcited electrons, and the resulting electron hole accepts an electron from H_2_O, leading to the formation of hydroxyl radicals. Oxygen accepts the electron, producing superoxide anions. Consequently, UV-irradiated TiO_2_ NPs have been demonstrated to induce oxidative stress due to these reactions (Fig. 4) (Marcone et al., 2012; Li et al., 2014b; Johnson et al., 2015; Horie and Tabei, 2021). The interaction of NPs with visible and UV light can also cause changes in their physicochemical properties. Studies have indicated that radiation-induced degradation of nanoparticle surfaces can lead to material transformations and increased aggregation over time (Horie and Tabei, 2021).

Additionally, the production of ROS during UV exposure can result in the release of toxic ions through material oxidation (Sun et al., 2014; Joo and Zhao, 2017). Clemente and colleagues observed a sublethal effect that varied depending on the nanoparticle form and lighting conditions. Pure anatase NPs caused more extensive oxidative damage under visible light, while the anatase-rutile mixture (80:20, m/m) exhibited a greater sublethal effect under UV light (Clemente et al., 2015). Uchino et al. found that under ultraviolet-A irradiation, the crystalline form of anatase generated a larger number of •OH in a dose-dependent manner compared to the rutile form, which showed less •OH generation (Uchino et al., 2002). Roy et al. reported that UVB pre-irradiation of TiO_2_ NPs caused more toxicity on freshwater algae because of the higher extracellular and intracellular ROS generation comparted to UVA pre-irradiation (Roy et al., 2020).

### 2.2 Silver nanoparticles

AgNPs are one of the most frequently manufactured and used nanomaterials, mainly due to their proven antifungal and antimicrobial effects (Kaweeteerawat et al., 2017). The estimated global consumption of AgNPs ranges from 320 to 500 tons per year, highlighting the potential release of AgNPs into the environment (Yu et al., 2013; Ferdous and Nemmar, 2020; Tortella et al., 2020; Ghobashy et al., 2021). The concentration of Ag in river water is typically below 5 ng/L (Peters et al., 2011; Wimmer et al., 2019), but in polluted areas, it can reach several hundred ng/L (Wimmer et al., 2019). Furthermore, accumulation of AgNPs in aquatic organisms from natural aquatic environments has been observed (Xiao et al., 2019). The fate and toxicity of AgNPs in the environment are influenced by various factors, including their aggregation, oxidation, dissolution, and potential transformation into sulfide or chlorinated forms. However, the exact amount and form in which AgNPs enter organisms, as well as the extent to which their toxicity is attributed to Ag^0^ NPs or released Ag^+^ ions, are still not fully understood (Ferdous and Nemmar, 2020; Tortella et al., 2020).

The behavior of AgNPs is also affected by their surface properties, as they can adsorb Ag^+^ ions and form colloids containing solid Ag^0^, free Ag^+^ or its complexes, and surface-adsorbed Ag^+^ (Lapresta-Fernandez et al., 2012; Zhang et al., 2018b; Zhao et al., 2021). The presence of organic matter and pH levels in the aquatic environment can further influence the interactions and behavior of AgNPs (Liu and Hurt, 2010b). It has been observed that AgNPs' toxicity is mainly dependent on their bioavailability, which is primarily determined by their size and degree of agglomeration, rather than their concentration alone (Pecoraro et al., 2017). Aging of AgNPs in the environment is also a factor that contributes to their toxicity, as prolonged exposure can lead to changes in their physicochemical properties and increased aggregation over time (Lekamge et al., 2019).

It is worth noting that AgNPs can release Ag^+^ ions under cellular conditions and interact with cellular components. The reactivity of Ag^+^ ions with biomolecules, particularly with Cys-containing proteins, plays a significant role in their toxicity (Wang et al., 2019b, 2020, 2021a). The transformation of AgNPs into Ag_2_S and silver thiolates has been observed in various organisms, including oysters and green algae, indicating the importance of these transformations in understanding the environmental fate and effects of AgNPs (Wang et al., 2016b; Zhang et al., 2020). The release of Ag^+^ ions and their subsequent impact on nuclear functions can induce endocrine disruptor-like effects in hepatocytes (Suarez et al., 2020). In many cases, Ag^+^ ions are found to be a major contributor of AgNP toxicity, because of their higher reactivity and bioavailability (Rajala et al., 2016; Ribeiro et al., 2017; Huang et al., 2018c; Khoshnamvand et al., 2020; Gao et al., 2021; Yan et al., 2021). However, other studies have suggested that the mechanisms of toxicity of AgNPs is due to the particles themselves (Bao et al., 2018; Carrazco-Quevedo et al., 2019; Wang et al., 2022). For example, AuNPs and TiO_2_ NPs, can induce toxic effects by triggering oxidative stress even without releasing ions, indicating a particle-specific effect (Girardello et al., 2016; Arini et al., 2020). The toxicity of AgNPs can vary depending on their coatings and forms. Studies have shown that different coatings, such as citrate, gum arabic, or PVP, can influence the toxicity and bioaccumulation of AgNPs in aquatic organisms (Farkas et al., 2011; Kwok et al., 2012). Chronic toxicity studies on organisms like *Daphnia* and fish have also demonstrated the potential adverse effects of AgNPs (Hou et al., 2017; Zhang et al., 2017). The interaction of AgNPs with aquatic organism can disrupt redox homeostasis, leading to oxidative stress and mitochondrial dysfunction, which can have detrimental effects on embryonic development and overall health (Khan et al., 2019; Lu et al., 2022). Oxidative stress induced by AgNPs was also reported in zebrafish brain tissues (Kokturk et al., 2022).

### 2.3 Zinc oxide nanoparticles

ZnO can be found in nature as the mineral zincite. ZnO NPs are widely used as photocatalytic materials due to their favorable properties, such as low cost, relatively low toxicity, chemical stability, and oxidation properties (Krezel and Maret, 2016; El-Bindary et al., 2019). The estimated concentrations of ZnO NPs in surface water and sewage treatment effluents, based on probabilistic material flow analysis, are approximately 13 ng/L and 430 ng/L, respectively (Gottschalk et al., 2009). Both the NPs themselves and the Zn^2+^ ions released from them are present in water systems. Therefore, when assessing the toxicity of ZnO NPs in the aquatic environment, it is necessary to consider the released ions as well. Mudunkotuwa et al. demonstrated that the solubility of ZnO NPs increases as their size decreases within the range of 4–130 nm (Mudunkotuwa et al., 2012).

The toxicity mechanism of ZnO NPs can also be attributed to their photocatalytic activity. Photo-induced ZnO has the ability to promote electron transfer from the filled valence band to an empty conduction band, producing electron-hole pairs that can migrate to the ZnO surface and be involved in redox reactions (Ong et al., 2018). Irradiation of ZnO NPs with blue light (400–500 nm) can promote the generation of ROS (Lipovsky et al., 2009). Some studies suggest that the toxicity of ZnO NPs is more induced by dissolved Zn^2+^ ions than by the particles alone (Deng et al., 2009; Blinova et al., 2010; Muller et al., 2010; Hou et al., 2018; Lee et al., 2021). However, other studies propose that the ability of ZnO NPs to generate ROS plays a major role in their toxicity (Ng et al., 2017). It was reported that NH_2_–ZnO NPs caused an accumulation of ROS in developing liver tissue, leading to the upregulation of autophagy-related genes and proteins. This cascade resulted in liver cell apoptosis and a reduction in liver size (Chen et al., 2022). Lin et al. found that ZnO NPs were more toxic than bulk ZnO or ionic zinc (ZnSO_4_·7H_2_O) to the crustacean *Daphnia pulex* (Lin et al., 2019). The higher toxicity of ZnO NPs compared to bulk ZnO is likely influenced by the combination of two factors: undissolved ZnO NPs have a large specific surface area with a high number of catalytically active centers, which leads to increased ROS formation, and ZnO NPs have higher solubility in water compared to larger ZnO particles, resulting in an increased concentration of free Zn^2+^ ions (Leitner and Sedmidubsky, 2016; Ng et al., 2017). Therefore, the solubility and dissolution rate of ZnO NPs in aqueous solutions are extremely significant in terms of their potential increased toxicity compared to bulk ZnO.

Studies have shown that ZnO NPs can affect the expression of proteins related to the hematological and immune systems in juvenile common carp (Chupani et al., 2017). The effects of acute and subacute exposures to ZnO NPs on zinc compartmentalization and biomarker expression have also been studied (Khosravi-Katuli et al., 2018). ZnO NPs were found to affect the expression of proteins related to cell motility and apoptosis in fish (Chupani et al., 2018). In a study by Bacchetta et al., *Daphnia magna* were exposed to 0.1 and 0.3 mg/L ∼50 nm ZnO NPs and 0.1 and 0.3 mg/L ZnSO_4_ for 21 days, and the ability of the organisms to regenerate was observed for another 21 days. Bacchetta et al. observed a reduction in the size of *D. magna* after the end of the ZnO NP exposure, which may be a sign of their impaired ability to regenerate (Bacchetta et al., 2017).

### 2.4 Gold nanoparticles

AuNPs can be found in nature in ore deposits. The formation of AuNPs is based on the abiogenic or bioorganic reduction of Au^+^/Au^3+^ complexes under changing physicochemical conditions. Reduction can occur on mineral surfaces by organic acids or during active/passive microbial biomineralization (Hough et al., 2011). AuNPs have long been considered highly stable in cells and the environment. However, recent studies have observed intracellular modifications of AuNPs (Balfourier et al., 2020). Balfourier et al. found re-crystallized gold as gold nanoleaves in fibroblasts after exposure to 4 nm AuNPs for up to 6 months. The authors hypothesized that AuNPs were dissolved within endolysosomes due to ROS and acidic pH. This hypothesis was supported by an experiment in which AuNPs were exposed to ROS generated in situ by electron beam-induced water radiolysis, confirming the dissolution of AuNPs in the presence of ROS and the potential for further re-crystallization of released Au ions as nanoleaves (Balfourier et al., 2020). In a study investigating the stability of citrate- and acrylate-coated AuNPs in the presence of naturally occurring humic acids, it was found that AuNPs in a medium with an ionic strength of 0.1 M rapidly and extensively aggregated. It can be concluded that in waters with high ionic strength (hard waters, estuaries), the aggregation will be significant, leading to sedimentation (Lapresta-Fernandez et al., 2012).

The effects of bioaccumulation of AuNPs in fish were studied in gilthead seabream (*Sparus aurata*) (Barreto et al., 2019), which concluded that the effects depend on the size, coating, and surface charge of the NPs. Detailed proteomic analysis of liver tissue in the same organism revealed different expression of 26 proteins induced after 96 h exposure to citrate- and PVP-coated 7 and 40 nm AuNPs. These up-regulated or down-regulated proteins are associated with the cytoskeleton and cell structure, gluconeogenesis, amino acid metabolism, energy metabolism, stress response, and various processes related to protein activity (protein synthesis, catabolism, folding, and transport) (Barreto et al., 2020). The effects of two forms of octahedral AuNPs were investigated in *Mytilus edulis* bivalves. The AuNPs induced ROS production and triggered the defense system (SOD, CAT, GST activities) depending on the NPs' physical form (Fkiri et al., 2018). In *Daphnia magna*, AuNPs tend to accumulate on the surface and in the gut, but no internalization was observed (Botha et al., 2016). In *Mytilus edulis* bivalves, it was found that the largest accumulation of AuNPs occurred in the area of the digestive glands, where lipid peroxidation was observed. Analysis of the gastrointestinal glands showed a decrease in protein thiols. Lysosomal membrane activity measured in the hemolymph was reduced (Tedesco et al., 2010b). However, only slight oxidative stress was observed using the same model organism and the same experimental conditions with different particle sizes (15 nm). The particles accumulated again in the digestive glands and the gills and mantle tissues (Tedesco et al., 2010a). The low enzymatic activity compared to the control group was also shown in the fish larvae *Macquaria ambigua* exposed to AuNPs (Shuster et al., 2021). The effects of AuNPs were also investigated in two other aquatic organisms: the freshwater clam *Corbicula fluminea* and the freshwater alga *Scenedesmus subspicatus*. These organisms were exposed to 10 nm amine-coated AuNPs. Accumulation of NPs in gills and digestive epithelium was observed. Their lysosomal localization led to a loss of surface stabilization of the amine, causing oxidative stress. The results showed that 20% mortality occurred when algae were exposed to the lowest concentration of AuNPs (1.6 × 10^2^ AuNPs/cell) used, while at the highest concentration (1.6 × 10^5^ AuNPs/cell), 50% mortality was observed (Renault et al., 2008).

### 2.5 Copper nanoparticles

Copper, as an essential trace micronutrient, plays a vital role as a co-factor in enzymatic and metabolic reactions, and it is also associated with oxygen transportation as part of hemocyanin in mollusks and crustaceans (Malhotra et al., 2020). Copper is a transition metal found in three oxidation states: Cu^0^ (solid metal state), Cu^+^ (cuprous ion), and Cu^2+^ (cupric ion). The various oxidation states of copper are used to design CuNPs with distinct properties. CuNPs have been widely used in various industries, including organic synthesis (catalysts) (Barot et al., 2016), pharmacy (drug delivery) (Wozniak-Budych et al., 2016), agriculture and food (antimicrobial agents) (Inkinen et al., 2017), and marine applications (paint and water treatment agents) (Ben-Sasson et al., 2016). In nature, copper monosulfide (CuS) occurs as the dark indigo blue mineral named covellite. Due to the low solubility of many sulfides in aqueous media, copper sulfide NPs with radii of 3–26 nm have been reported in oxygenated waters (Rozan et al., 2000). The toxicity of copper in the aquatic environment strongly depends on its speciation profile, and therefore, evaluating the forms of copper ions released into the aquatic environment is necessary to analyze their toxicity and bioavailability. Syed and Coombs studied copper metabolism in fish and mammals and showed that copper is mainly accumulated in the gills or lungs, kidneys, brain, and liver (Syed, 1982). Many studies have shown that copper imbalance is linked to specific genetic diseases (Menkes and Alzheimer's diseases) in humans and that high copper concentrations are toxic to both humans and fish, causing morphological, physiological, and biochemical effects (Malhotra et al., 2020).

As mentioned earlier, high levels of copper can cause abnormalities in living organisms. Non-degradable CuNPs commonly used in various applications have the potential to accumulate in the environment. There is plenty of evidence of the toxicity of CuNPs in the aquatic environment, which tend to release Cu^2+^ ions, leading to the formation of hydroxyl free radicals and subsequent damage to the membranes they interact with (Keller et al., 2017; Wang et al., 2019c; Johari et al., 2020). For example, it was demonstrated that CuNPs and Cu^2+^ cause intestinal developmental defects by inducing endoplasmic reticular and ROS stress in zebrafish (Zhao et al., 2020). A comparison of the toxicity of CuNPs and ionic copper (hemato/hepatotoxicity) was investigated in *Cyprinus carpio* (Noureen et al., 2018), concluding that the NPs were more toxic, inducing an inflammatory solid response and necrosis. Exposure of *Danio rerio* embryos to CuNPs caused developmental abnormalities and increased mortality (Aksakal and Ciltas, 2019). Another study focused on serum parameters in the fish *Oreochromis niloticus*, finding unusually high levels of urea nitrogen and creatinine, confirming potential hazardous effects (Canli et al., 2018). The toxicity of CuNPs was also investigated in the crustacean *Daphnia magna* (Lu et al., 2017) and the bivalve *Corbicula fluminea* (Koehle-Divo et al., 2019), indicating a dose-dependent impact and dependency on nanoparticle size. Another study showed that the toxicity decreased with increasing pH of the NPs and was concentration-dependent (Xiao et al., 2018).

In summary, the behavior and toxicity of metallic nanoparticles in the aquatic environment are influenced by various factors, including their physicochemical properties, transformations, interactions with natural colloids, and exposure conditions. Understanding these factors is crucial for assessing the potential environmental risks associated with metallic nanoparticles and developing effective risk management strategies.

## 3 Biomarkers of oxidative stress in aquatic organisms

The balance between oxidant factors and antioxidant defenses (enzymatic or nonenzymatic) can be used to estimate the toxic effects induced by metallic NPs. The use of biomarkers of oxidative stress to determine the level of oxidative stress in an exposed organism is of great importance in environmental toxicology studies. These biomarkers typically include antioxidants, products of oxidative stress, reactive nitrogen species (RNS) and free oxygen radicals. In several recent ecotoxicity studies, various biomarkers have been analyzed to assess the extent of oxidative stress caused by different metallic NPs (Khan et al., 2017b; Valerio-Garcia et al., 2017; Abdelazim et al., 2018; Huang et al., 2018b; Tang et al., 2019; Renuka et al., 2020; Lu et al., 2022; Sekar et al., 2023). For instance, 2′,7′-dichlorodihydrofluorescein diacetate (DCFH-DA) and dihydroethidium (DHE) are commonly utilized to measure ROS (Lu et al., 2022). Flow cytometry is a powerful tool for measuring intracellular ROS levels with these probes.

**Fig. 5:**
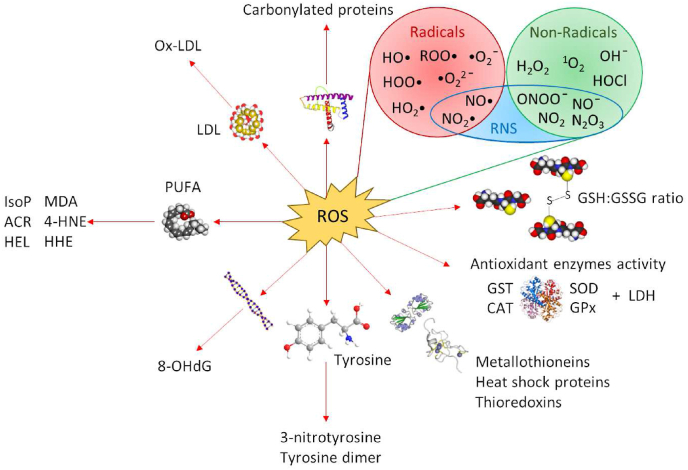
The classification and formation pathways of selected biomarkers of oxidative stress. Reactive oxygen/nitrogen species (ROS/RNS) can interact with low-density lipoprotein (LDL), DNA, tyrosine and proteins resulting in products as oxidized-LDL (Ox-LDL), 8-hydroxy-2-deoxyguanosine (8-OHdG), 3-nitrotyrosine, tyrosine dimer and carbonylated proteins. Biomarkers of peroxidation of polyunsaturated fatty acids (PUFA) includes malondialdehyde (MDA), 4-hydroxy 2-nonenal (4-HNE), 4-hydroxyhexenal (HHE), hexanoyl-lysine adduct (HEL), acrolein (ACR), isoprostanes (IsoP). Antioxidant enzymes and molecules includes superoxide dismutase (SOD), catalase (CAT), glutathione peroxidase (GPx), glutathione S-transferase (GST), reduced and oxidized glutathione (GSH/GSSG). Other stress biomarkers include lactate dehydrogenase (LDH), metallothioneins, heat shock proteins and thioredoxins.

Glutathione metabolism-related markers are usually analyzed together with lipid peroxidation measured by malondialdehyde (MDA) concentration and some other enzymes. The most important enzymes that are part of antioxidant mechanisms include NADPH-dependent flavoenzymes: SOD, GPx, and CAT. The enzyme SOD occurs in both the cytosol and mitochondria. The antioxidant activity of SOD is based on the catalysis of the transformation of superoxide radicals to oxygen and hydrogen peroxide, which is further reduced by CAT or GPx, which, in addition to H_2_O_2_, also reduces lipid peroxides (Fig. 2) (Lushchak, 2014b). Other antioxidant enzymes include GST, GR, the thioredoxin/thioredoxin reductase system, and others (Bernard et al., 2015). However, insufficient enzymatic defense is known to reduce cell hydroxyl radical levels. The only defense organisms have against this radical is the presence of low molecular mass substances, including glutathione, proline, α-tocopherol, carotenoids, and flavonoids (Bernard et al., 2015). In the case of toxic metal pollution, low-molecular-weight and Cys-rich proteins called metallothioneins (MTs) are also used as oxidative stress biomarkers. They act as strong chelators of thiophilic metal ions and play a role in the detoxification mechanisms of aquatic organisms by scavenging oxygen free radicals and binding metals (Andrews, 2000; Cavaletto et al., 2002). The selection of common biomarkers of oxidative stress is demonstrated on Fig. 5.

The damaging effects of oxidative stress on tissues and cellular components induced by metallic NPs have become a significant topic in aquatic environmental toxicology studies. This knowledge about oxidative damage has prompted the extension of environmental and ecotoxicological studies to precisely detect oxidative stress in aquatic organisms. Furthermore, studies based on oxidative biomarkers that indicate oxidative stress can be helpful in large-scale environmental monitoring programs. Therefore, this chapter mainly focuses on recent findings regarding these beneficial biomarkers, as well as their classification and applicability (Table 1).

**Table 1:** Summary of oxidative stress biomarkers in assessing the effect of metallic NPs on aquatic organisms.

Nanoparticles (NPs)	NPs (coating)/chemical	NPs' size (nm)	Test species/sample	Concentration (mg/L)	Time Exposure	LC50	Studied effects	Method (oxidative stress)	Stress biomarkers	Results	Notes/conclusion	References
AgNPs	cit-AgNPs (citrate coated)	5–10	*Oncorhynchus mykiss* (rainbow trout) gill cells	0.1; 1.0; 5.0 and 10	48 h	–	Ag uptake, cytotoxicity, oxidative stress, transepithelial resistance	Fluorescent probe (monochlorobimane - mBCl)	Depletion of GSH	GSH increase at exposure levels of 0.1; 1.0 and 5.0 mg/L of cit-AgNPs and Ag^+^, while PVP-AgNPs caused elevated levels at concentrations 1–10 mg/L	ICP-MS used to measure uptake of NPs	Farkas et al., 2011
	PVP-AgNPs (polyvinylpyrrolidone)	7 (avg)		0.1; 1.0; 5.0 and 10	48 h	–						
	Ag^+^ ions (AgNO_3_)	salt		0.1; 1.0; 5.0 and 10	48 h	–						
	AgNPs (amine as capping agent)	17.78 ± 12.12	*Labeo Rohita* (gills and liver)	10; 20; 30; 45 and 55	28 days	–	Oxidative stress, lipid peroxidation, Micronucleus test, Comet assay	GST (Habig et al., 1974), GSH (Jollow et al., 1974), MDA (Wilchek and Bayer, 1990)	GST, GSH and MDA	Sharp decline in the activities of GST and this decrease of activity increase the MDA content, the level of GSH increased	AgNPs are genotoxic in nature and produce micronuclei, comet cells and induces oxidative stress	Khan et al., 2017
	AgNPs	25	*Danio rerio* (Zebrafish) adult	8; 45 and 70 μg/L	30 days	–	Bioaccumulation of AgNPs, oxidative stress, histological changes, gene expression	IHC, Western-Blotting, PCR	MT-1	MT-1 increased in gill and liver	The histological analysis showed different degrees of toxicity on the gills as well as huge necrosis in the intestine. No lesion was detected in the liver.	Pecoraro et al., 2017
	PVP/PEI (polyethylenimine)-coated AgNPs	5.08 ± 2.03	*Danio rerio* (liver and intestine)	Dietary exposure to Brine shrimps larvae previously exposed to 100 ng and 100 μg/L AgNPs	21 days	–	Ag bioaccumulation in the food chain, gene transcription profiles, the lysosomal membrane stability, histological analysis	Microarray analysis, qPCR	MT-2, GST, genes involved in protein ubiquitination	176 genes were found to be significantly regulated in liver after 21 days of exposure. Lipid transport and lipid localization, cellular response to chemical stimulus and response to xenobiotic stimulus were significantly regulated. GSH metabolism was significantly affected	Dietary exposure resulted in Ag transfer from the brine shrimps exposed to the AgNPs' suspension to zebrafish	Lacave et al., 2017
	AgNPs dispersed in PVP	11.95 ± 5.3	*Chapalichthys pardalis* adult (liver, gills, and muscle)	1.93 and 4.08	21 days	10.32 mg/L (96 h)	Oxidative stress, macromolecular and metabolic biomarkers	Measuring absorbance: CAT (Radi et al., 1991), SOD (Crapo et al., 1978), GPx (Lei et al., 1995), TBARS (Buege and Aust, 1978), Carbonylated proteins (Levine et al., 1994), macromolecules (Lowry et al., 1951; Zöllner and Kirsch, 1962, Dubois et al., 1951), lactate level	SOD, CAT, GPx, thiobarbituric acid reactive species (TBARS), protein oxidation, macromolecules (proteins, lipids, and carbohydrates), and metabolites (glucose and lactate)	AgNPs produce oxidative stress in *C. pardalis* adults, as evidenced by a diminution in antioxidant enzymes activity and an increase in TBARS and oxidized proteins.	–	Valerio-García et al., 2017
	Laminarin based AgNPs (L-AgNPs)	N/A	*Danio rerio* adult (liver, gills and brain)	12.61	14 days	25.22 mg/L (96 h)	Ag uptake, cytological changes, oxidative stress, histological analysis	The spectrophotometric methods	SOD, CAT, GPx	The activities of SOD, CAT and GPx were decreased significantly in the C-AgNPs exposed while L-AgNPs showed no significant difference when compared to control.	–	Renuka et al., 2020
	Commercial AgNPs (C-AgNPs)	<100		9.96	14 days	19.93 mg/L (96 h)						
	AgNPs powder (99% pure) and AgNO_3_	49.6 (avg)	*Oryzia latipes*, 4–5-month-old (liver)	1.0 and 25 μg/L	1, 2, 4 and 10 days	34.6 ± 0.9 μg/L (AgNPs), 36.5 ± 1.8 μg/L (AgNO_3_) (96 h)	Changes in the expression of stress-related genes	Real time RT-PCR method	GST, MT, HSP 70, p53, CYP 1A and transferrin gene	In overall, AgNPs caused a higher stress response compared to AgNO_3_	Mechanisms of toxicity of the AgNPs is distinguishable from AgNO_3_. Cellular damage, DNA damage or carcinogenic and oxidative stresses were more connected to AgNPs whereas inflammatory responses and metallic detoxification processes to AgNO_3_.	Chae et al., 2009
	Commercial AgNPs and AgNO_3_	10(N_10_); 35(N_35_) and 600–1600 (N_bulk_)	*Oncorhynchus mykiss* (gills, liver, kidneys)	10 and 100 μg/L N_10_, 10 and 100 μg/L N_35_, 100 μg/L N_Bulk_, and 0.1 μg/L AgNO_3_	10 days	–	Ag uptake, lipid peroxidation, gene expression, histological analyses	Lipid peroxidation (MDA, TBARS assay), Real-time PCR	MDA, genes cyp1a2, cyp3a45, hsp70a, gpx, and g6pd	No observed lipid peroxidation in any of the tissues analyzed for any of the silver particles tested, However, expression of cyp1a2 in the gills after exposure to N_10_.	–	Scown et al., 2010
	Curcumin-coated AgNPs (C-AgNPs), tyrosine-coated AgNPs (T-AgNPs) and epigallocatechin gallate-coated AgNPs (E-AgNPs)	10.56 ± 2.27 (T-AgNP), 9.27 ± 1.29 (E-AgNP), 13.68 ± 0.76 (C–AgNP)	*Paratya australiensis*	30 μg/L	96 h	–	The stability of differently coated AgNPs the impact of NP aging on potential toxicity, lipid peroxidation, oxidative stress	CAT (Hadwan, 2016), GST (Habig et al., 1974), TBARS	CAT, GST, MDA	Except for aged C-AgNPs, the levels of MDA in exposed shrimp, whether to fresh or aged AgNPs, did not display significant differences compared to the control. However, aged C-AgNPs notably raised MDA levels compared to their fresh form and the other treatment groups. This could be linked to more pronounced oxidative stress, likely due to an increased release of free Ag^+^ ions from C-AgNPs compared to T-AgNPs and E-AgNPs.	Elevated LPO and CAT levels detected *in P. australiensis* pointed to the presence of oxidative stress, implying the shrimp's vulnerability to AgNP exposure. The heightened oxidative stress observed in shrimp subjected to aged C-AgNPs implies that the aging process of AgNPs influences their biological reactions.	Lekamge et al., 2019
	AgNPs (synthesized) and AgNO3 (Sigma/commercial?)	50	*Oreochromis mossambicus* (liver, gill, brain)	0, 25, 50, 75 and 100 μg/L AgNPs and AgNO3	7 days	–	Oxidative stress, neurotoxicity, immune parameters and histological changes in gill	AAS (Gobi et al., 2018), Oxidative stress (Lowry et al., 1951; Haghighat et al., 2021; Buege and Aust, 1978; Reznick and Packer, 1994), Antioxidant parameters (Suzuki, 2000; Cohen et al., 19710; Gopi et al., 2019; Rotruck et al., 1973), Neurotoxicity (Ellman et al., 1961); Non-specific immune parameters (Stolen et al., 1992; Kumari and Sahoo, 2006); Histology: (Hawkins et al., 2015)	LPO, PCA, GST, GPx, SOD, CAT, MT, LYZ, MPO, RBA, Ache	The study found that higher concentrations of AgNO3 enhanced antioxidant enzyme activities in fish gills more effectively than AgNPs. Both substances caused gill damage, including telangiectasia and epithelial cell hyperplasia. Increasing concentrations of AgNPs and AgNO3 led to silver accumulation, causing oxidative stress and changes in enzymatic and non-enzymatic parameters, resulting in cellular damage.	The study revealed that silver bioaccumulation in organisms depends heavily on environmental conditions. Silver accumulation leads to oxidative stress, causing cellular damage that increases with higher concentrations of AgNPs and AgNO3. Enzymes like SOD, CAT, and GPx initially counteract ROS but eventually fail due to toxicity. Non-enzymatic defenses like MT and GSH also become ineffective, disrupting immune parameters. AgNPs and AgNO3 also cause neurotoxicity by penetrating brain cells. Continuous exposure to these pollutants can harm the survival, development, and reproduction of organisms, particularly the freshwater fish *O. mossambicus*.	Sibiya et al., 2022
	AgNPs	47,68	Polypedates macultas (blood, tissue, liver, kidney)	1, 5 and 10 mg/L	60 days	–	Bioaccumulation, morphological abnormalities, blood profiles, immunological markers, biochemical markers, oxidative stress:	Marklund and Marklund, 1974; Aebi, 1984	LPO, SOD, CAT	Antioxidant levels (SOD and CAT) significantly decreased in the treatment groups. This indicates that Ag/ZnO-NPs are toxic to aquatic organisms and amphibians even at sub-lethal concentrations. The species *P. maculatus* can serve as a bioindicator for nanomaterial contamination in freshwater systems.	Higher concentrations of ZnO-NPs (50 mg/L) and Ag-NPs (10 mg/L) negatively impacted haematology, metabolism, immunity, and the antioxidant system. Ag and Zn accumulated in tissues, causing oxidative stress and toxicity. High levels of Ag/ZnO-NPs increased liver (ALT, AST, ALP) and kidney (urea, creatinine) damage biomarkers while reducing antioxidant enzymes (SOD, CAT). Low-level exposure (1 mg/L) initially boosted biomarkers, indicating stress adaptation, but overall health declined at higher concentrations.	Murthy et al., 2022
	AgNPs (citrate coated, nanocomposix)	20	*Lymnaea stagnalis and Planorbarius corneus (hepatopancreas, stomach, hemolymph, different tissues)*	10, 50, 100, 250 and 500 μg/L	7 days	–	Bioaccumulation, oxidative stress. Immune responses	Commercial kits, Microplate Reader with fluorescence excitation at 485 nm and emission at 520 nm	ROS, SOD, CAT, MDA, GST, AChe	The hemolymph was more susceptible to oxidative stress from AgNPs than the hepatopancreas, as indicated by increased lipid peroxidation (malondialdehyde formation). Neurotoxicity was observed in *L. stagnalis* at high concentrations (500 μg/L).	The hepatopancreas and stomach showed a significant increase of accumulation from 50 μg/L AgNPs on, which was not the case in the other studied tissues (kidney, mantle and foot). As for the biological response, the hemolymph of *L. stagnalis* and *P. corneus* are more susceptible to AgNP exposure-induced oxidative stress compared to the hepatopancreas. Although no lipid damage was found in the hepatopancreas, neurotoxicity was observed in this tissue. Furthermore, although the effects produced by Ag + should not be underestimated, the Ag particulate form better explained the biological responses, indicating that the NPs themselves play a significant role in exerting toxicity.	Wang et al., 2022
	Ag^+^			11.96 μg/L								
	Biogenic AgNPs	33	*Cyprinus carpio* (gills, liver, muscle)	0.184, 0.276, and 0.552 mg/L (1/15th, 1/10th, and 1/5th according to 96 h LC50)	28	LC50: 2.36 mg/L (96 h 2, 4, 6, 8, and 10 mg/L)	Haematological alterations, oxidative stress, histological changes, differential gene expression patterns, and bioaccumulation	RT qPCR, Antioxidant activity (Lowry et al., 1951), SOD (Marklund and Marklund, 1974), CAT (Claiborne, 1985), GPx (Rotruck et al., 1973), GST (Habig et al., 1974), GSH (Moron et al., 1979),	SOD, CAT, GPx1a, GST-α, CYP1A, and Nrf-2	The highest Ag accumulation was in the gills. Changes in haematological parameters were noted. Inhibition or stimulation was noted of SOD, CAT, GPx, GST, and GSH. Antioxidant defence system of *C. carpio* were downregulated. Downregulation of oxidative stress-related genes: Cu–Zn SOD, CAT, GPx1a, GST-α, CYP1A, and Nrf-2, affects their expression patterns.	In this study, biogenic silver nanoparticles (AgNPs) were synthesized through the reaction of ionic silver (AgNO₃) with an aqueous extract of onion peel (Allium cepa L).	Sekar et al., 2023
	AgNPs coated PVP	70–80	*Dreissenna bugensis*	2, 10 and 50 μg/L	96 h	–	Toxicity of different structures (sphere, cube and prism) of AgNPs	Spectrophotometric assay and fluorescence probe methodology	AChe, LPO (TBARS, H_2_O_2_, COX)	Prismatic nAg most potently reduced acetylcholinesterase (AChE) activity and increased viscosity, while also altering protein aggregation patterns. All nAg shapes decreased AChE reaction fractal dimension, but spheric nAg had the most pronounced effect. In contrast, dissolved silver showed no impact. These findings underscore the importance of nanoparticle geometry in determining aquatic toxicity.	–	Auclair et al., 2022
	Ag + ions (AgNO_3_)			2 and 10 μg/L								
TiO_2_ NPs	TiO_2_ anatase	5	*Daphnia magna*	1.0 and 10.0	24 h	10.5 mg/L (48 h)	bioconcentration factor (BCF), biomagnification factor (BMF), metabolic profile, histology	GC-MS	Bioinformatic analysis of determined metabolites	GSH-metabolism significantly disturbed	The results show that the acute toxicity, BCF, and BMF all decreased with increasing size or rutile content of nTiO2.	Chen et al., 2019
	TiO_2_ anatase	10				13.2 mg/L (48 h)						
	TiO_2_ anatase	100				37.0 mg/L (48 h)						
	TiO_2_ 80%anatase, 20%rutile	20				28.4 mg/L (48 h)						
	TiO_2_ rutile	25				60.7 mg/L (48 h)						
	TiO_2_ rutile	25	*Danio rerio* embryo and adult	10; 50; 100	7 days	–	Oxidative stress	ELISA, qRT-PCR	SOD, CAT and GST activity	The activities of CAT, SOD and GSTs decreased in gills, liver compared to control. The activities of antioxidant enzymes in intestine tissue were not significantly affected by TiO_2_ NPs but were increased under high concentration of TiO_2_ NPs.	Results confirmed by showing up-regulation of genes for SOD/CAT/GST by qRT-PCR	Tang et al., 2019
	TiO_2_	10–15	*Ruditapes decussatus* (gills and digestive gland)	50 and 100 μg/L	14 days	–	Metal accumulation, biochemical and behavioral responses	The spectrophotometric methods: SOD (McCord and Fridovich, 1969), CAT (Aebi, 1984), GST (Habig et al., 1974), MDA (Buege and Aust, 1978), AChE (Ellman et al., 1961)	SOD, CAT, GST, AChE activity, MDA, nitric oxide	SOD, CAT, GST and AChE activities were induced in gills and digestives gland in concentration and nanocomposite type dependent manner. Higher MDA levels in gills and digestive gland after Au–TiO_2_ exposure than TiO_2_ NPs	Decoration of NPs could increase the toxicity of these chemicals in aquatic organisms.	Saidani et al., 2019
	Au–TiO_2_	10–15										
	TiO_2_	25 ± 5	*Mytilus coruscus* (gills and digestive glands)	2.5; 10.0	14 days	–	Oxidative stress	SOD: the nitro blue tetrazolium (NBT) method (Beauchamp and Fridovich, 1971), the spectrophotometric methods: CAT (Góth, 1991), GPx (Lawrence and Burk, 1976), GST (Habig et al., 1974), GSH (Ringwood et al., 1999), MDA (Ohkawa et al., 1979)	SOD, CAT, GR, GPx, GST activity, GSH and MDA	An increase in MDA level and a decrease in SOD and GSH activities were observed in gill of mussels exposed to 10 mg/L TiO_2_ NPs. SOD, CAT and GSH levels increased in the digestive gland.	Gills are more sensitive to these stressors as compared with digestive glands.	Huang et al., 2018
	TiO_2_ + inorganic lead Pb or Pb(II) (coexposure)	21	*Astyanax serratus* (liver and blood)	0.5; 5.0 and 50 ng/g	96 h (intraperitoneal injection)	–	Oxidative stress, genotoxicity: the Comet assay, DNA diffusion assay and piscine micronucleus test	The spectrophotometric methods: SOD (Crouch et al., 1981), CAT (Aebi, 1984), GST (Keen et al., 1976), MT-1 (Viarengo et al., 1997)	SOD, CAT and GST activity, MT-1	GST increased in all co-exposed groups whereas SOD was inhibited. CAT activity increased significantly in the highest co-exposure group. MTs increased significantly in co-exposed group Pb(II) + 0.5 ng/g TiO_2_ NPs	–	Delmond et al., 2019
	TiO_2_ anatase	<25 nm	*Danio rerio* embryos	0, 1.0, 10 and 100 mg/L under Vis or a combination of Vis and UV light	96 h	–	Acute toxicity, sublethal parameters, oxidative stress	The spectrophotometric methods: CAT (Aebi, 1984), GST (Keen et al., 1976), acid phosphate (AP, dos Prazeres et al., 2004)	CAT, GSH, AP	Alterations in the activities of CAT and GST were determined. No clear dose-response relationship was observed. However, effects on biochemical biomarkers CAT, GST and AP were dependent on both the type of TiO_2_ and illumination condition	Low acute toxicity to the embryos of *D.rerio* was observed. The mortality rate for the anatase/rutile mixture was higher compared to pure anatase TiO_2_ at 100 mg/L. The rate and sublethal effects were increased under UV.	Clemente et al., 2013
	TiO_2_ 20% rutile, 80% anatase	25 nm										
	TiO_2_ anatase	<25 nm	*Piaractus mesopotamicus* (liver, gill, brain, blood)	100 mg/L under Vis or a combination of Vis and UV light	21 days	–	Oxidative stress, genotoxicity: the Comet assay	The spectrophotometric methods (SOD, CAT, GST, AP activity) and Comet assay	lipid hydroperoxide (LPO), carbonylated proteins (PCO), SOD, CAT and GST. acid phosphatase (AP), Na^+^, K^+^-ATPase and MT	Oxidative stress was dependent on crystal phase and illumination condition. Under Vis light, exposure to TiO_2_ anatase NPs increased CAT activity, presence of PCO and genetic damage (caused more oxidative damage without co-exposure to UV). Exposure to anatase:rutile mixture showed protein carbonylation, genetic damages and a higher GST activity when co-exposed to UV light.	No fish mortality has been observed including lack of Ti accumulation in muscle tissue. On other hand, sublethal effects were observed in dependence crystal phase and illumination condition.	Clemente et al., 2015
	TiO_2_ 20% rutile, 80% anatase	25 nm										
	TiO_2_ 86% anatase, 14% rutile + Suwannee River natural organic matter (SRNOM) - (coexposure)	25.1 ± 8.2 nm	*Daphnia manga* and *D.rerio* larvae	0, 0.1, 0.25, 0.5, 0.75, 1, 2.5, and 5 mg/L for *D. magna*, and 0, 5, 10, 50, 100, 150, 200, and 250 mg/L for *D.rerio* larvae under simulated solar radiation (SSR) conditions	48 h	>250 mg/L for both species with and without the presence of SRNOM (under laboratory condition) For Daphnia magna, phototoxic LC50 values were 1.03 mg/L without SRNOM, 0.26 mg/L with 5 mg/L SRNOM For *D.rerio* larvae, phototoxic LC50 values were 39.9 mg/L and 26.3 mg/L, with or without the presence of 5 mg/L SRNOM, respectively.	Influences of SRNOM on the phototoxicity of TiO_2_ NPs under relevant solar radiation conditions (TiO_2_ particle stabilization, UV attenuation, and photosensitization/ROS quenching), lipid peroxidation	Fluorescence probes for ROS (Setsukinai et al., 2003; TBARS assay (lipid peroxidation)	ROS, MDA	The presence of SRNOM showed quenching effects (ROS, MDA) on TiO_2_ NPs in an aquatic system dependent on test species, with D. magna being the more sensitive species.	–	Li et al., 2016
	Anatase TiO_2_ + NOM + UV (coexposure)	<25 nm	*Daphnia magna* neonates (<24 h, second brood)	0, 0.5, 1, 1.5, and 2 mg/L TiO_2_ NPs + 1, 2 and 4 mg/L NOM	48 h	–	Photo-induced toxicity, UV attenuation, ROS quenching	Fluorescence assay for ROS	ROS	ROS production was significantly reduced in a NOM concentration–dependent manner. NOM in concentration 4 mg/L reduced anatase TiO_2_ NPs toxicity by nearly 100%.	NOM (4 mg/L) attenuated UV by <10% in the exposure system. However, the authors suggest the reduction of anatase TiO_2_ NPs toxicity by NOM is based on an ROS quenching mechanism	Wormington et al., 2017
	TiO_2_	10	*Unio ravoisieri* (gill, digestive glands)	10, 100, 1000 μg/L	48 h and 7 days	–	Oxidative stress	H_2_O_2_ (Biomagreb kit), CAT (Aebi, 1984; Beutler, 1984), MDA and AChe (Ellman et al., 1961)	H_2_O_2_, CAT, MDA, AChe	TiO_2_ NPs elevated oxidative stress in both gills and digestive glands of the freshwater mussel, as indicated by increased CAT activity, MDA, and H_2_O_2_ levels. These effects were concentration-dependent. Additionally, AChe inhibition suggests disruption of the nervous system at TiO_2_ concentrations exceeding 1 mg/L.	TiO_2_ NPs caused a progressive decline in acetylcholinesterase (AChE) activity. These findings suggest that TiO_2_ NPs generate free oxygen radicals and neurotoxic byproducts.	Smii et al., 2021
	TiO_2_ and different carbon (C) allotropes (C–NHS (carbon-based nanohybrids); CNT (carbon nanotube); CNF (carbon nanofiber))	1045–4698	*Daphnia magna* (adult)	7.8, 15.6, 31.25, 62.5, 125, 250 mg/L	48 h	TiO_2_ NPs (94.05 mg/L)TiO_2_–CNF (42.27 mg/L) TiO_2_-CNT (28.34 mg/L)	Oxidative stress (TiO_2_- conjugated carbon nanofiber (CNF), and TiO_2_-conjugated multi-walled carbon nanotube (CNT))	commercially available kits with using a standard microplate reader (absorbances and fluorescence)	SOD, CAT, GST	TiO_2_/C-NHs exhibited low acute toxicity to Daphnia magna but caused behavioural changes and bioaccumulation within the organism. Microscopic analysis confirmed nanoparticle uptake. Increased antioxidant enzyme activity at higher concentrations indicated oxidative stress induced by the nanohybrids.	Microscopy confirmed nanoparticle uptake and accumulation in the gut. These findings suggest that TiO_2_/C-NHs pose a potential risk to freshwater ecosystems.	Malatjie et al., 2022
	TiO_2_	20–70	*Danio rerio and Carassius gibelio* (hemocytes, liver and gill)	*Danio rerio* 10 mg/L and *Carassius gibelio* 260 mg/g (in food)	8 days	–	Oxidative, proteolytic, genotoxic and apoptotic	ROS (Patetsini et al., 2013) MDA (Niehaus and Samuelsson, 1968)	ROS, MDA	TiO_2_ nanoparticles (NPs) negatively impacted animal physiology and swimming behavior across species and tissues. A conserved mechanism appears to underlie these effects, involving immune system activation, oxidative stress, lysosomal damage, protein and lipid oxidation, DNA damage, and eventual apoptosis through ubiquitin-mediated proteolysis.	After exposure to TiO_2_-NPs, a common mechanism is activated. This mechanism involves immune system stimulation, resulting in ROS production. It also leads to lysosomal membrane damage, protein carbonylation, lipid peroxidation, DNA damage, proteolysis by ubiquitin, and ultimately apoptosis	Bobori et al., 2020
	TiO_2_ with ammonia (total ammonia nitrogen - TAN)	25	*Danio rerio* (adult female, gill and liver)	TAN 0, 3 and 30 mg/L TiO_2_-NPs 0, 0.1 and 1 mg/L	8 weeks	–	Toxicity of co-exposure, oxidative and antioxidant	Beutler and Kelly (1963), Ohkawa et al. (1979), Aebi (1984), Drotar et al. (1985), Paoletti et al. (1986), Hou et al. (2015) and Armenteros et al. (2009)	GSH, MDA, CAT, GPx, SOD, GST, PC (protein carbonylation)	TiO_2_ NPs exacerbated ammonia toxicity in fish. Both substances independently increased gill and liver ammonia levels, inducing oxidative stress by depleting antioxidant defenses through Nrf2-Keap1 pathway inhibition. However, their combined effects were synergistic, leading to more severe tissue damage and a pronounced imbalance between oxidants and antioxidants. These findings highlight the significant environmental risk posed by the combined presence of ammonia and n-TiO_2_.	Combined exposure to ammonia and nano-TiO_2_ intensified oxidative stress and tissue damage in zebrafish gills and livers. Both substances individually disrupted the antioxidant system by inhibiting Nrf2-Keap1 signaling. The presence of nano-TiO_2_ exacerbated ammonia-induced toxicity, potentially by enhancing ammonia uptake or damaging cell membranes. This synergistic interaction resulted in severe oxidative imbalance. Notably, even low, non-toxic concentrations of ammonia or TiO_2_ NPs could adversely affect fish health over time.	Guo et al., 2022
ZnO NPs	ZnO	30 ± 5	*Nile Tilapia* (muscles)	1.0 and 2.0	7 and 15 days	–	Stress markers	mRNA expression levels by RT-PCR	SOD, CAT, GR, GPX, GST activity, GSH and MDA concentration	Decreased compared to control group	–	Abdelazim et al., 2018
	ZnO	50	*Carassius auratus* (gills and livers)	10 and 100 μg	7; 14 and 21 days	–	Antioxidant enzyme activity, lipid peroxidation, histopathology	The spectrophotometric methods: SOD (Sun et al., 1988), CAT (Aebi, 1984), GST (Habig et al., 1974), MDA (Uchiyama and Mihara, 1978)	CAT, SOD a GST activity, MDA content	Increase in CAT and SOD activity in the gills and livers, especially after 14 days of exposure. Increase of GST was observed in gills after 7 days and in livers after 14 days. The levels of MDA in the livers increased after 7 days.	–	Benavides et al., 2016
	ZnO	25 (28)	*Cyprinus carpio* juvenile	0, 50, 500 mg of ZnO NPs/kg of feed	6 weeks + 2 week recovery period	–	Haematology, biochemical analysis, zinc accumulation, histopathology, lipid peroxidation	TBARS assay	MDA	The level of lipid peroxidation significantly decreased in fish of treated groups after two weeks of recovery	No significant Zn uptake in any of tested organs. During exposure, a histological analysis showed slight histopathological alterations in the kidneys. After the recovery period, the higher exposure group exhibited a significant (p < 0.05) increase in aspartate aminotransferase activity and a significant (p < 0.05) decrease in alanine transferase activity	Chupani et al., 2018
	Commercial ZnO and ZnSO_4_	30	Caspian roach *(Rutilus rutilus caspicus)*	**Scenario A:** 0, 10, 20, 40, 50, 80 mg/L of ZnO NPs for 4 days **Scenario B:** 48 mg/L of ZnO NPs and ZnSO_4_ for 4 days (acute exposure) and 4.8 mg/L of ZnO NPs and ZnSO4 for 28 days (sub-acute exposure). **Scenario C:** tested organisms were transferred to clean water to allow depuration to occur for 14 days otherwise conditions same as Scenario B.	1, 2,3, 4 and 28 days	24 h (78 ± 7 mg/L), 48 h (61 ± 5 mg/L), 72 h (53 ± 6), and 96 h (48 ± 3 mg/L)	The acute and sub-acute effects (the concentration-response curves and LC_50_), zinc accumulation, antioxidant response and stress blood parameters, histopathological effects, Zn compartmentalization	SOD (Winterbourn et al., 1975; CAT Aebi, 1984, GST (Habig et al., 1974), GSH (Tietze, 1969), MDA (Satoh, 1978, and protein content (Bradford, 1976)	SOD, CAT, GST, LDH, GSH, MDA, protein content	After the treatments, the activity of biomarkers was significantly increased for both ZnO NPs and ZnSO_4_. However, during the depuration phase, their values returned to background levels. Most of the effects were observed at acute concentrations (48 mg/L; 4 days) and in the presence of ZnSO_4_.	The gills showed the highest accumulation of Zn after sub-acute exposure (4.8 mg/L; 28 days), followed by the liver, kidney, and muscle. Among these organs, the gills, liver, and muscle exhibited higher concentrations of Zn from ZnO NPs. During the depuration period (14 days), Zn content in each organ decreased, but complete removal only happened in the muscle. Histopathological examinations revealed that the exposure to ZnO nanoparticles (NPs) led to increased lesions in the gill, liver, and kidney. The severity of these alterations was directly proportional to the amount of Zn accumulated in the respective organs. During the depuration phase, the lesions partially regressed for both ZnO NPs and ZnSO_4_, but full recovery did not occur.	Khosravi-Katuli et al., 2018
	ZnO NPs, bulk ZnO and ZnSO_4·_7H_2_O	<100 nm	*Daphnia pulex*	1/5th of the 24 h EC_50_	24 h and 48 h	**EC**_**50**_**(24h):** 0.32 mg/L (ZnO NPs), 0.76 mg/L (bulk ZnO) and 0.98 mg/L (ZnSO_4·_7H_2_O) **EC**_**50**_**(48h):** 0.19 mg/L (ZnO NPs), 0.46 mg/L (bulk ZnO) and 0.50 mg/L (ZnSO_4·_7H_2_O)	Proteomic analysis, genetic analysis	SCX Fractionation and LC-MS/MS (iTRAQ), q-PCR analysis)	Up and down-regulated proteins	In all three treatments, there was an upregulation of vitellogenins fused with SOD, peroxiredoxin, and GST. Additionally, several differentially expressed proteins were associated with ER stress, suggesting the involvement of ER stress in the observed toxicity.	All forms of Zn had similar effects on *D. pulex*, leading to the downregulation of Chitinase expression, disruption of Ca^2+^ homeostasis, and reduction in digestive enzyme expression. The ZnO NP treatment specifically showed the expression of 29 proteins that were not observed in other treatments. Among them, histone (H3) and ribosomal proteins (L13) were significantly affected by the ZnO NP treatment. Interestingly, increased expression levels of H3 and L13 genes were not exclusively induced by the ZnO NP treatment but also exhibited sensitivity to Zn ions at the same exposure concentration. These findings suggest that the three zinc substances have a similar mode of action, and the release of zinc ions is the primary contributor to ZnO NP toxicity to *D. pulex* at low concentrations.	Lin et al., 2019
	ZnO	41,96	*Polypedates macultas* (blood, tissue, liver, kidney)	1, 10 and 50 mg/L	60 days	–	Bioaccumulation, morphological abnormalities, blood profiles, immunological markers, biochemical markers, oxidative stress:	Marklund and Marklund, 1974; Aebi, 1984	LPO, SOD, CAT	Antioxidant levels (SOD and CAT) significantly decreased in the treatment groups. This indicates that Ag/ZnO-NPs are toxic to aquatic organisms and amphibians even at sub-lethal concentrations. The species *P. maculatus* can serve as a bioindicator for nanomaterial contamination in freshwater systems.	Higher concentrations of ZnO-NPs (50 mg/L) and Ag-NPs (10 mg/L) negatively impacted haematology, metabolism, immunity, and the antioxidant system. Ag and Zn accumulated in tissues, causing oxidative stress and toxicity. High levels of Ag/ZnO-NPs increased liver (ALT, AST, ALP) and kidney (urea, creatinine) damage biomarkers while reducing antioxidant enzymes (SOD, CAT). Low-level exposure (1 mg/L) initially boosted biomarkers, indicating stress adaptation, but overall health declined at higher concentrations.	Murthy et al., 2022
	Bio-functionalized ZnO	–	*Cyprinus carpio* (gill, liver, muscle)	0.382, 0.573 and 1.146 mg/L	28 days	Not observed (96 h LC50, concentrations of 0, 2, 4, 6, 8 and 10 mg/L)	Antioxidant defense mechanisms, histomorphology, and oxidative stress	RT qPCR, Antioxidant activity (Lowry et al., 1951), SOD (Marklund and Marklund, 1974), CAT (Claiborne, 1985), GPx (Rotruck et al., 1973), GST (Habig et al., 1974), GSH (Moron et al., 1979),	ROS, SOD, CAT, GPx, GST, GHS	Altered hematological parameters and increased ROS, elevating activities of SOD, CAT, GPx, GST, and GSH. Histopathological analysis showed lamellar fusion, aneurysm, cytoplasmic vacuolation, nuclear alteration, necrotic muscle fiber, and pyknotic nuclei in gills, liver, and muscles. Up-regulated expressions of SOD1, CAT, GPx1a, GST-α, CYP1A, and Nrf-2 genes. Zn bioaccumulation was highest in gills, followed by liver and muscle.	Biogenic nanomaterials are less toxic than the conventional approach.	Sekar et al., 2023
	ZnO	50	*Takifugu obscurus -* juveniles (2 months old) (gill, kidney and liver)	0, 10, 20, 50, 100, and 200 mg/L	96 h	–	Protective effects of salinity to ZnO	kits provided by Nanjing Jiancheng Bioengineering Engineering Research Institute (Nanjing, China)	MDA, CAT, SOD, GSH	Short-term exposure to ZnO NPs can induce oxidative stress, reduce survival rates, and disrupt osmoregulatory function in juvenile *T. obscurus*. However, increased salinity appears to counteract the toxic effects of ZnO NPs through two potential mechanisms: promoting aggregation and limiting ion release, as well as enhancing osmotic adjustment	Higher salinity (15 ppt) promotes ZnO NP aggregation, limiting metal ion release and reducing their bioavailability. Elevated salinity alleviates ZnO NP toxicity through osmotic adjustment via Na+/K ± ATPase activation.	Lin et al., 2024
	ZnO	50	*Takifugu obscurus* (early life stages, including embryos, early hatched larvae, and juveniles), (gill, kidney, and intestine)	0, 10, 20, 40, 60, and 80 mg/L	96 h	–	Toxicity	kits provided by Nanjing Jiancheng Bioengineering Engineering Research Institute (Nanjing, China)	MDA, GSH, SOD, CAT	ZnO NPs had detrimental effects on *T. obscurus* at all life stages. Embryos exhibited reduced hatching rates and surface abnormalities at higher NP concentrations. Hatchlings displayed deformities, and post-hatching survival was significantly compromised. Juvenile survival declined consistently with increasing NP exposure. Biochemical analyses revealed oxidative stress, evidenced by elevated MDA levels and decreased GSH, SOD, and CAT activities in multiple tissues.	The observed decline in hatching, larval, and juvenile survival rates of *T. obscurus* with increasing ZnO nanoparticle (NP) concentration is primarily attributed to the toxicity of the nanoparticles themselves, with a secondary contribution from released Zn_2_+ ions. ZnO NPs induced significant oxidative stress across multiple tissues.	Tang et al., 2024
AuNPs	Au (two octahedra forms)	10–200	*Ruditapes decussatus* clams	0.1 and 1.0	14 days	–	Antioxidant enzyme activity, lipid peroxidation and determination of vitellogenin -like proteins	The spectrophotometric methods: SOD (Marklund and Marklund, 1974), CAT (Aebi, 1984), GST (Habig et al., 1974), MDA (Buege and Aust, 1978)	CAT, SOD and GST activity, MDA content	Au_0.03 and Au_0.045 exposure causes oxidative stress in form and concentration dependent manner	–	Fkiri et al., 2018
	cit-AuNPs and PVP-AuNPs coating + a pharmaceutical drug gemfibrozil (GEM)	40	*Sparus aurata* (liver, gills, muscle and brain)	9 experimental conditions: 4, 80 and 1600 μg/L cit-AuNPs and PVP-AuNPs; 150 μg/L GEM; mixture of 150 μg/L GEM with 80 μg/L cit-AuNPs and PVP-AuNPs	96 h	–	Swimming performance, antioxidant defenses, Au content, LPO, ROS, oxidative stress	ChE (Ellman et al., 1961); CAT (Claiborne, 1985); GR (Carlberg and Mannervik, 1975); GPx (Athar and Iqbal, 1998); non-protein thiols (NPT) (Parvez et al., 2003; GST (Habig et al., 1974; LPO levels (Ohkawa et al., 1979)	GST, CAT, GPx and GR; ChE activity, NPT. LPO (TBARS)	Both exposure to AuNPs resulted in the induction of antioxidant defenses in the liver and gills.	The size, coating, surface charge, and aggregation/agglomeration state of nanoparticles were found to be determining factors influencing the accumulation and effects of AuNPs. The presence of PVP coating enhanced the stability of AuNPs in artificial seawater, leading to an increase in their bioavailability and subsequent accumulation in fish tissues. AuNPs and GEM combined effects in gills were generally low in liver, they were higher than the predicted.	Barreto et al., 2019
	cit-AuNPs and PVP-AuNPs	7 and 40	*Sparus aurata* (liver)	80 μg/L	96 h	–	Total gold content and bioaccumulation factor, proteomic analysis	Protein identification and functional analysis by MALDI-TOF/TOF	Stress related proteins	In liver cells, AuNPs had varying impacts on multiple metabolic pathways. The heightened presence of proteins linked to energy metabolism (such as ATP synthase subunit beta), stress response (like 94 kDa glucose-regulated protein), and the structural integrity of the cytoskeleton (including actins and tubulins) might indicate the initial indications of oxidative stress triggered by AuNPs.	Even though the liver of *S. aurata* exposed to 7 nm PVP-AuNPs exhibited increased gold accumulation, the 7 nm cit-AuNPs demonstrated higher bioactivity, leading to more pronounced effects on the liver proteome.	Barreto et al., 2020
	AuNPs	5.3 ± 1	*Mytilus edulis* (digestive gland, gill, and mantle)	750 ppb AuNP and 0,2 mM CdCl_2_	24 h	–	Oxidative stress, lipid peroxidation, lysosomal membrane stability, metal determination	Thiol-containing proteins (1DE and 2DE SDS/PAGE), lipid peroxidation (Shaw et al., 2004), lysosomal membrane stability (Regoli et al., 2004)	Thiol-containing proteins, MDA content	The primary site of AuNP accumulation in *M. edulis was* the digestive gland, which also showed elevated lipid peroxidation levels. Furthermore, decreased thiol-containing proteins was determined in AuNPs exposed group. The measurements of lysosomal membrane stability in hemolymph indicated reduced values in both treatments, with AuNPs demonstrating higher stability. Oxidative stress was observed within the initial 24 h of exposure to AuNPs.	–	Tedesco et al., 2010
	cit-AuNPs	∼15	*Mytilus edulis* (digestive gland, gill and mantle)	750 ppb cit-AuNPs (GNP, gold 0); 1 mM menadione and GNP plus 1 mM menadione	24 h	–	Metal determination, oxidative stress, lipid peroxidation, thiol-containing proteins	Thiol-containing proteins (1DE and 2DE SDS/PAGE), lipid peroxidation (Shaw et al., 2004); spectra analysis: thioredoxin reductase activity (Arnér et al., 1999) and GSH/GSSG ratio (Hissin and Hilf, 1976)	Thioredoxin reductase, Thiol-containing proteins, MDA levels, GSH, GSSG	The findings indicate that exposure to AuNPs leads to a moderate degree of oxidative stress, significant enough to oxidize thiols in glutathione and proteins, yet without triggering lipid peroxidation or inducing an increase in thioredoxin reductase activity.	The primary accumulation site for AuNPs is the digestive gland, with minor amounts also detected in the gill and mantle through ICP-OES analysis. AuNPs did lead to a reduction in protein thiol levels, indicating the targeting of protein thiols by ROS under the specific conditions employed.	Tedesco et al., 2010
	Au/TiO_2_	20 (gold core 10 nm surrounded TiO_2_ NPs 25 nm)	*Unio ravoisieri* (gill, digestive glands)	100 and 200 μg/L	48 h and 7 days	–	Oxidative stress	CAT (Aebi, 1984), MDA and TBARS (Haque et al., 2019), GST (Habig et al., 1974), GSH, Ache	CAT, MDA, TBARS, GST, GSH, Ache, H_2_O_2_	Exposure to Au/TiO_2_ NPs induced oxidative stress in both gills and digestive glands of the freshwater mussel. Increased levels of hydrogen peroxide, CAT, GSH, and MDA indicated oxidative damage, while AChe activity was inhibited, suggesting disruption of the nervous system. Mussel behavior was also affected, with reduced clearance rates.	–	Dellali et al., 2021
	AuNPs	5 and 50	*Macquaria ambigua* (one-day old)	0.05 μM gold (AuCL3); 5 nm - 5 μM; 50 nm - 50 μM	6 days	–	Oxidative stress	Aebi (1984), Smith et al. (1988), Drotar et al. (1985),	CAT, GR, GSSG, GST, GSH,	Larvae exhibited minimal oxidative stress response to all gold treatments. Gold uptake varied by form: aqueous gold and 5 nm AuNPs readily entered cells, while 50 nm AuNPs were primarily taken up through the mouth or gills. Cellular uptake of aqueous gold resulted in intracellular nanoparticle formation, whereas 5 nm AuNPs aggregated within cells. AuNP exposure negatively impacted golden perch larvae, with effects dependent on nanoparticle size.	Gold exposure led to yolk sac edema, most pronounced in larvae exposed to 50 μM of 5 nm AuNPs, which exhibited yolk sacs 1.5 times larger than controls and notochord bending. These larvae experienced 100% mortality within two days. Other gold-exposed groups showed less than 25% mortality, suggesting potential adaptation to gold exposure.	Shuster et al., 2021
	amine-coated AuNPs	10	*Corbicula fluminea*	1.6 × 10^3^, 1.6 × 10^4^ and 1.6 × 10^5^ AuNPs/cell	7 days	–	Bioaccumulation, molecular impact, gene expression, oxidative stress	Real-time RT-PCR, AAS	Genes for MT, CAT, SOD, GST, subunit 1 of the cytochrome-C-oxidase, RNA12s	Research has shown that they have the potential to induce excessive production of MTs and provoke oxidative stress in the gills.	The contamination of bivalves highlighted the nanoparticles' capability to accumulate within the organisms and enter both gill and digestive epithelial tissues. Their localization within lysosomes resulted in the degradation of their coating, initiating oxidative stress.	Renault et al., 2008
CuNPs	CuNPs and CrNPs + their mixture	–	*Daphnia magna*	0; 0.4; 2; 10; 50 and 100 μg/L for CuNPs	48 h and 21 days	0.63 mg/L (CuNPs, 48 h)	Antioxidant enzyme activity and Acetylcholinesterase activity (AChE)	AChE (Ellman et al., 1961), the spectrophotometric methods: SOD (Marklund and Marklund, 1974), CAT (Goth, 1991), GST (Habig and Jakoby, 1981)	AChE, CAT, SOD and GST activity	Across all measured parameters, it was evident that CuNPs exhibited greater toxicity compared to CrNPs. The biochemical reactions displayed heightened GST activity and reduced AChE activity. Additionally, SOD and CAT activities increased at lower concentrations but decreased at higher concentrations in all exposure scenarios.	Variations in concentration yielded discernible alterations in all assessed biomarkers (AChE, SOD, CAT, and GST). Stress-induced effects on the physiological functions of *D. magna* were evident due to the presence of CuNPs and its combinations with CrNPs at microgram-per-liter levels.	Lu et al., 2017
	CuNPs	10–30	*Takifugu fasciatus* (liver)	0; 20 and 100 μg/L	30 days	–	Cu content, oxidative stress, apoptosis and immune response	Oxidative stress analyses used kits. (Xu et al., 2009; Cheng et al., 2018)	MDA, SOD, CAT, GSH, activities of caspases, succinate dehydrogenase (SDH), Na^+^-K^+^-ATPase and cytochrome *c*, HSP90 and HSP70	The elevation in CuNPs dose led to noteworthy increases in MDA concentration, SOD and CAT activities, as well as GSH concentration in the liver, in comparison to the control group. These observations suggest that: (i) exposure to CuNPs can intensify ROS production in *T. fasciatus*'s liver, thereby resulting in liver damage evidenced by cell apoptosis; and (ii) following CuNPs exposure, antioxidant defense system was stimulated to counteract the detrimental impacts of ROS.	The results of this study suggest that in juvenile *T. fasciatus*, CuNPs triggered liver apoptosis through the caspase-dependent pathway mediated by mitochondria, along with activation of the p53-Bax-Bcl2 pathway. The liver's antioxidant and immune defense mechanisms were activated as a response to shield cells from oxidative stress and apoptosis. However, these defense mechanisms were not entirely successful in safeguarding the entire fish organism when exposed to severe oxidative conditions.	Wang et al., 2019
	CuO NPs	≤50	*Danio rerio* embryos	0,5; 1 and 1,5	96 h	–	Fish embryo acute toxicity test, Cu content, oxidative stress and immune system	RT-PCR	Metal transcription factor 1 (*mtf-1*), Heat shock protein 70 (*hsp70)*, Nuclear factor kβ (*nfkb*), Interleukin-1 (*il-1β*), CCAAT/enhancer binding protein (C/EBP)β (*cebp*), Transferrin (*trf*), Toll like receptor4 (*tlr-4*), Toll like receptor22, Actin (*tlr-22*)	The findings from this study demonstrated that CuO NPs induced developmental toxicity, oxidative stress and immunotoxicity in *D.rerio* embryos. In the CuO NPs treatment groups, the mRNA expressions of *mtf-1* were observed to be reduced compared to the control group. Conversely, exposure to CuO NPs notably elevated the mRNA expression of *hsp70* and nfkb genes when compared to the control group. RT-PCR results also showed that the transcription of *il-1β, tlr-4, tlr-22, trf, cebp* was changed by the application of CuO NPs.	The analysis indicated that while CuO NPs couldn't penetrate the tissues of *D.rerio* embryos/larvae (Raman spectroscopy), they still led to elevated mortality rates, delayed hatching, and reduced heartbeat rates.	Aksakal and Ciltas, 2019
	CuNPs	32 ± 1	*Rainbow trout (hepatocytes)*	323 μg/L	48 h	990 ± 150 μg/L	Co-exposure MPs and CuNPs on regulating oxidative stress	qPCR	CAT, SOD, GPx, GST	CuNPs and dissolved Cu increased CAT and SOD gene expression, indicating oxidative stress in hepatocytes. CuNPs also upregulated ATP1A1. Microplastics had no significant impact, alone or combined with CuNPs. These findings suggest that acute Cu exposure induces oxidative stress in hepatocytes, independent of microplastic presence.	Both CuNPs and dissolved copper ions elevated oxidative stress gene expression. Dissolved copper appears to be the primary driver of this response. Microplastics alone had no significant effect on gene expression. While microplastics can adsorb copper, this adsorption did not alter the copper-induced changes in gene transcripts.	Razmara et al., 2023
	Cu^2+^			8 μg/L		–						
	Microplastics (MPs)	4–6 μm		2 mg/L		–						
	CuNPs	24	*Pangasianodon hypophthalmus* (liver, kidney, and gill tissues)	3.0, 3.3, 3.6, 3.9, and 4.2 mg/L	96 h	3.85 mg/L (96 h)	Oxidative stress, neurotransmission, and cellular metabolism	Misra and Fridovich, 1972, Takahara et al., 1960, Habig et al., 1974; Paglia and Valentine, 1967; Uchiyama and Mihara, 1978	SOD, CAT, GST, GPx, Ache, LPO	Cu and Cu-NPs significantly increased oxidative stress markers (CAT, SOD, GST, GPx, LPO) in liver, gills, and kidneys. Acetylcholinesterase activity in the brain was inhibited. Moreover, both Cu forms induced cellular metabolic stress, evidenced by elevated cortisol, HSP70, and blood glucose levels.	High Cu and CuNPs concentrations (9.0 and 4.2 mg/L, respectively) elevated oxidative stress markers (CAT, SOD, GST, GPx, LPO) in liver, gills, and kidneys. Additionally, these concentrations increased protein (ALT, AST) and carbohydrate (LDH, MDH) metabolic enzymes in these organs. Both Cu forms induced significant biochemical and metabolic disturbances, causing liver and gill tissue damage after 96 h.	Kumar et al., 2023
	Cu			7.0, 7.5, 8.0, 8.5, and 9.0 mg/L		8.04 mg/L (96 h)						
	CuNPs	40	*Procambarus clarkii*	0.2, 0.5, 1, 2, 5 and 10 mg/L	28 days	1.18 mg/L (72 h)	Toxicity and bioaccumulation	kits provided by Nanjing Jiancheng Bioengineering Engineering Research Institute (Nanjing, China)	SOD, CAT and GSH	Crayfish accumulated significantly more copper from CuSO_4_ than CuNPs, primarily in gills, followed by hepatopancreas and muscle. CuNP exposure decreased antioxidant enzyme activity after 48 h, indicating oxidative stress. However, hepatopancreas histology and crayfish growth remained unaffected. These findings suggest that CuNPs induce oxidative stress in crayfish primarily through released copper ions, with gills as the main accumulation site.	CuNPs released copper ions in freshwater and formed aggregates, with stability increasing at higher concentrations. CuSO4 caused hepatopancreas morphological damage, CuNPs did not induce significant histological changes. While CuNP exposure did not inhibit crayfish growth over 28 days, the observed toxicity was primarily attributed to released copper ions rather than the nanoparticle form itself.	Yang et al., 2022
	CuSO_4_			positive control		0.54 mg/L (72 h)						
	CuO NPs	32.84	Labeo rohita (gill)	70.79 and 117.99 mg/L	15, 30 and 45 days	353.98 mg/L (96 h)	Accumulation, oxidative stress, genotoxicity	Maehly and Chance (1954); Gatoo et al. (2014); Gatta et al. (2000)	CAT, TBARS, LPO	CuO-NPs bioaccumulated more at higher concentrations (1/3rd 96 h LC50 was significantly higher compared to 1/5th of 96-h LC50 of CuO-NPs). Catalase activity decreased while lipid peroxidation increased, indicating oxidative stress. The highest rates of DNA damage occurred at the highest concentration after 45 days. These effects worsened over time and with increasing CuO-NP concentration. Overall, sublethal CuO-NP exposure caused metal overload, oxidative stress, and genotoxicity.	High CuO-NP concentrations are lethal to fish, while sublethal levels cause bioaccumulation, oxidative stress, and genotoxicity.	Aziz and Abdullah, 2023

### 3.1 Glutathione metabolism-related enzymes

GSH is a tripeptide composed of glutamic acid (with γ-peptide bond), cysteine, and glycine. In addition to its antioxidant functions, GSH also has a detoxifying function, specifically a conjugative function, and serves as a precursor of cysteine. GSH is a relatively efficient ligand for metal ions due to its intracellular abundance (Krezel and Bal, 1999, 2004). For example, zinc ions can interact with GSH and affect GSH metabolism through efficient binding (Krezel and Bal, 2004). Oxidized glutathione (GSSG) can also serve as a ligand for Zn^2+^, forming complexes with comparable affinity and stability as GSH (Krezel and Bal, 1999; Krezel et al., 2011). Both forms of glutathione also form significant complexes with other metal ions, including Ag^+^ ions (Leung et al., 2013). GSH is mainly found in the cytosol and mitochondria of cells. One mechanism of GSH's antioxidant function is the formation of the GSSG dimer from the GSH monomer while removing ROS. Another antioxidant feature of GSH is its ability to restore ascorbic acid (vitamin C), one of the antioxidants. The level of oxidative stress is often determined by the ratio of oxidized GSSG dimer to reduced GSH monomer. This ratio reflects the rate of depletion of this antioxidant and thus the functionality of the antioxidant defense against oxidative stress (Lushchak, 2014b; Zhang et al., 2021). The crucial biomarkers of oxidative stress are antioxidant enzymes including CAT, GPx, SOD and GST. These enzymes play a significant role in neutralizing ROS and protecting cells from oxidative damage. An increase or decrease in their activity can signal oxidative stress.

**Fig. 6:**
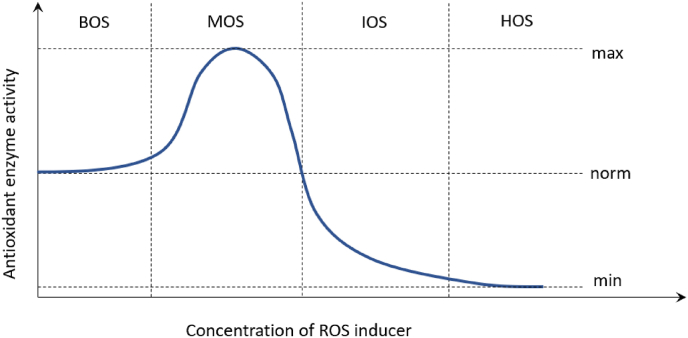
Nonlinear behavior of enzyme activity in response to the intensity of oxidative stress. The enzyme activity is normal in the first zone of low (basal) intensity oxidative stress (BOS). In the second zone of mild oxidative stress, the enzyme activity is elevated to the maximum level, dropping eventually in the zone of intermediate stress level (IOS) and tending toward the minimum in the zone of high oxidative stress (HOS).

Several studies related to the oxidative stress in fish caused by metallic NPs were conducted. The stress biomarkers provided insights into the oxidative stress levels induced by exposure to AgNPs in freshwater fish, highlighting the potential impact on cellular health and function. The enzymatic antioxidant activities were activated in response to oxidative stress caused by Ag accumulation. The exposure to AgNPs also led to elevated levels of lipid peroxidation and protein carbonyl activity (Sibiya et al., 2022a). The concentration dependent alteration of GST, GR and CAT was found in the gills and liver of carp fish (Kakakhel et al., 2024). Downregulation in the expression patterns of oxidative stress genes of SOD, CAT, GPx, GST was determined in carp fish treated with biogenic synthesized AgNPs (Sekar et al., 2023). Additionally, exposure to AgNPs led to the accumulation of ROS and MDA, inhibition of enzyme activities, and downregulation of genes associated with mitochondrial function and apoptosis. These results indicate that AgNP exposure can induce oxidative stress, mitochondrial dysfunction, and toxicity in zebrafish embryos (Khan et al., 2019). A significant decline in GST activities was observed in AgNPs treated freshwater fish *Labeo rohita* (Khan et al., 2017a), while the levels of GSH were increased significantly (Khan et al., 2017b). GSH depletion was detected in rainbow trout (*Oncorhynchus mykiss*) gill cells after exposure to citrate and PVP-coated manufactured AgNPs and AgNO_3_ (Farkas et al., 2011). Decrease of GSH was also demonstrated in crayfish exposed to CuNPs and CuSO_4_. This was likely because GSH was used up to bind the excessive copper ions, which helped alleviate oxidative stress in exposed crayfish. These findings suggest that the toxicity of CuNPs could be attributed to the effects of copper ions (Yang et al., 2022). An extended set of biomarkers was examined in *Oreochromis niloticus* muscles exposed to ZnO NPs. Reduced GSH concentration and SOD, CAT, and GST activity declined in the dose and time-dependent exposure manner (Abdelazim et al., 2018). Indications of oxidative stress as a main cause of toxicity, with decreased levels of antioxidant enzymes SOD and CAT has been also found in tadpoles exposed to AgNPs and ZnO NPs (Murthy et al., 2022). Furthermore, increased ROS production in a dose-dependent manner and SOD decline was reported in crucian carp exposed to ZnO NPs (Hong et al., 2022). The content of GSH and the activities of the SOD and CAT decreased in juveniles Takifugu obscurus exposed to ZnO NPs, suggesting that the antioxidant responses were suppressed (Lin et al., 2024; Tang et al., 2024). On the other hand, a contradictory result was obtained in a study conducted in *Carassius auratus* exposed to ZnO NPs, where activity of the same enzymes increased significantly (Benavides et al., 2016). The increased activity of SOD, CAT, and GST has been also determined in the liver, gills, and kidney of *Pangasianodon hypophthalmus* after exposure to ZnNPs (Kumar et al., 2020). These findings can be explained by the non-linear behavior of these markers, as described by Lushchak (2016) (Fig. 6).

According to this concept, the activity of antioxidant enzymes increases during the phase of mildly increased stressor dose or concentration. After passing a threshold value, the activity decreases back to the basal level and beyond, indicating the depletion of the antioxidant.

Several studies have recorded elevated antioxidant enzymes levels in the various bivalves, indicating a dependency on the size and concentration of TiO_2_ NPs. Altered levels of antioxidant enzymes were observed in the clam *Ruditapes decussatus,* scallop *Chlamys farreri*, and the mussels *Unio tumidus*, *Unio ravoisieri*, *Mytilus galloprovincialis* and *Mytilus coruscus* after exposure to TiO_2_ NPs (Canesi et al., 2010; Xia et al., 2017; Huang et al., 2018b; Marisa et al., 2018; Gnatyshyna et al., 2019; Saidani et al., 2019; Smii et al., 2021). In a study performed on the bivalve clam *Ruditapes decussatus* the activity of SOD and GST in gills decreased, yet the CAT activity increased significantly. Even more, distinct results were obtained for enzyme activities in the digestive gland in concentration-dependent manner (Saidani et al., 2019). The activity of these enzymes in the same species resulted in contrasting outcomes when the comparison was made between different physical forms of AuNPs of the same size (Fkiri et al., 2018). Exposure to AuNPs and TiO_2_ NPs caused oxidative stress in the gills and digestive glands of bivalve *Unio ravoisieri*. There was a significant increase in the activities of CAT and GST, as well as in MDA content, depending on the concentration of AuNPs and TiO_2_ NPs and the specific organ examined (Dellali et al., 2021). The activity of SOD and GST remained unchanged in another freshwater bivalve (Vale et al., 2014), where TiO_2_ NPs were used together with free Cd^2+^, and no effect of TiO_2_ presence was observed. Contrasting results of the same enzyme's activity were obtained (Huang et al., 2018b) at two different pH levels of seawater (pH 8.1 and pH 7.3), confirming that acidity modulates the impact of NPs. The above-mentioned enzyme activities were compared between marine rotifer and crustaceans exposed to metal-polluted seawater (Jeong et al., 2019). In both cases, the most significant effect was observed on the GST activity.

Exposure to TiO_2_ NPs conjugated with various carbon allotropes increased the activity of SOD, GST, and CAT in *Daphnia magna* (Malatjie et al., 2022). TiO_2_ NPs have been shown to induce oxidative stress in fish, as demonstrated by the exposure of juvenile olive flounder (*Paralichthys olivaceus*) to a TiO_2_ NPs in concentration 1 and 10 mg/mL (Huang et al., 2018a). A significant, concentration-dependent decrease in SOD, CAT, and GST activity in different tissues of adult zebrafish exposed to 25 nm TiO_2_ rutile NPs was shown in gill and liver tissue (Tang et al., 2019). Cr, Fe, and Ni doped TiO_2_ NPs resulted in significant changes in antioxidant activities of SOD, CAT, GPx and lipid peroxidation in the gills and intestines of both species the juvenile common carp and juvenile goldfish (Pirsaheb et al., 2019).

### 3.2 Metallothionein as a stress marker

Metallothioneins (MTs) can also be included as thiol biomarkers of oxidative stress. MTs are proteins with high cysteine content, responsible for neutralizing hydroxyl radicals from both essential and non-essential metals, bioaccumulation of toxic metals and detoxification, homeostatic regulation of Zn and Cu, and regulation of cell proliferation and apoptosis (Ruttkay-Nedecky et al., 2013; Wang et al., 2014; Rodrigo et al., 2020; Krezel and Maret, 2021). MT was used as a biomarker of metal pollution in fish exposed to elevated zinc and copper levels, resulting in a significant correlation between metal exposure and hepatic expression of MT (M'Kandawire et al., 2017) and as a biomarker of AgNPs toxicity (Pecoraro et al., 2017). Another study compared the toxic effect of ionic silver and AgNPs in the fish *Oryzias latipes*, confirming the high sensitivity of MT gene expression in reaction to both pollutants (Chae et al., 2009). In addition, the effect of AgNPs on lysosomes in oyster embryos has been observed, which is associated with destabilization and increased metallothionein mRNA levels in adult individuals (Ringwood et al., 2010). Triggering of MT overproduction was observed in the gills and visceral mass of *Corbicula fluminea* after exposure to AuNPs (Renault et al., 2008). In comparison with control, decreased thiol-containing protein levels have been reported in *M. edulis* after exposure to AgNPs, which is probably connected with the oxidation of thiol-groups by ROS (Tedesco et al., 2010a).

### 3.3 Other biomarkers of oxidative stress

Besides GSH and enzymes related to ROS scavenging, there are some other frequently used biomarkers of oxidative stress. The most common is MDA that is a biomarker of lipid peroxidation in which ROS attack polyunsaturated fatty acids in cell membranes. High levels of MDA are reliable indicator of oxidative damage to lipids. Together with SOD, CAT, and GST, MDA comprises the most often repeated set of markers. The level of these markers was studied in Nile tilapia muscles on the 7th and 15th day of 1 and 2 mg/L ZnO NP treatments. Results showed that MDA concentration significantly increased in groups exposed to ZnO NPs (Abdelazim et al., 2018). On the contrary, significant disturbances in lipid peroxidation parameters were not observed in common carp juvenile dietary exposed to ZnO NPs for six weeks (Chupani et al., 2018). That result is inconsistent with a study from Hao et al., which determined high lipid peroxidation levels in the livers and gills of carps after 50 mg/L ZnO NP-waterborne treatment for 30 days (Hao et al., 2013). Other studies have also reported increased amounts of MDA after exposure to ZnO NPs (Benavides et al., 2016; Nadhman et al., 2016) or other NPs (Ates et al., 2013; Wang et al., 2016c; Khan et al., 2017b; Saidani et al., 2019; Bobori et al., 2020; Chen et al., 2021; Auclair et al., 2022). Among other studied stress markers belong reactive aldehydes products such as 4-hydroxynonenal (4-HNE) (Lapresta-Fernandez et al., 2012) and acrolein (Li et al., 2008). Furthermore, the determination of carbonylated proteins (Tamarit et al., 2012; Sibiya et al., 2022a), protein ubiquitin, or thioredoxins (Lapresta-Fernandez et al., 2012) is used for monitoring oxidative stress. The thioredoxin/thioredoxin reductase system indicates oxidative damage that affects sulfur-containing amino acids. Decreased levels of thioredoxins indicate increased oxidative stress (Lapresta-Fernandez et al., 2012). Another representative of the protein stress marker is tyrosine, respectively its dimer, dityrosine (Vazquez et al., 2019). Furthermore, heat shock proteins are a group of proteins that are produced in response to stress conditions, including oxidative stress. These proteins help in the proper folding and repair of damaged proteins. Increased expression of HSPs is a biomarker of cellular stress (Chae et al., 2009; Kumar et al., 2023; Rangaswamy et al., 2024). Other biomarkers of oxidative stress are DNA damage products, oxidized bases, and adducts with lipid peroxidation products. The most determined marker is 8-oxo-deoxyguanosine and 8-hydroxy-2′-deoxyguanosine (8-OHdG) (Valavanidis et al., 2009; Siddiqi et al., 2012; Omari Shekaftik and Nasirzadeh, 2021). Non-specific markers of cell damage by oxidative stress include the determination of the enzyme lactate dehydrogenase (LDH) released during cell lysis. A different level of LDH (p < 0.05) in serum from *Oreochromis niloticus* between control and 7-days-exposed variants with 10 mg/L suspensions of 80–100 nm ZnO NPs has been demonstrated. At the same time, no significant differences (p > 0.05) were found on the 14th day using the same particles and conditions. No significant differences were also observed on the 7th and 14th using 80–100 nm ZnO NPs in the concentration of 1 mg/L or 40–60 nm ZnO NPs in 1 mg/L and 10 mg/L concentration (Kaya et al., 2017). Similarly, significant toxicity based on LDH-level has not been shown in the study of Lee et al., in which carps were exposed to 0.1, 0.3, 0.8, and 2.4 mg/L ZnO NPs (<100 nm) for 12 weeks (Lee et al., 2014). On the contrary, serum LDH rates have been observed after 8-days-exposure of *Oncorhynchus mykiss* to 0.2–0.4 mg/L suspensions of AgNPs (Imani et al., 2015). Oxidative damage in aquatic organisms can also be observed based on lysosomal membrane stability, mitochondrial damage, inhibition of metabolic activity, and loss of membrane integrity (Lapresta-Fernandez et al., 2012).

In summary, biomarkers of oxidative stress play a crucial role in assessing the impact of metallic NPs on aquatic organisms (Table 1). Glutathione metabolism-related markers, antioxidant enzyme activities, lipid peroxidation products, metallothioneins, and other oxidative stress biomarkers provide valuable insights into the level of oxidative stress and the potential toxic effects induced by metallic NPs. These biomarkers can be useful for large-scale environmental monitoring programs and furthering our understanding of oxidative damage in aquatic organisms.

## 4 Overview of techniques used to determine oxidative stress biomarkers level in aquatic organisms exposed to metallic nanoparticles

**Fig. 7:**
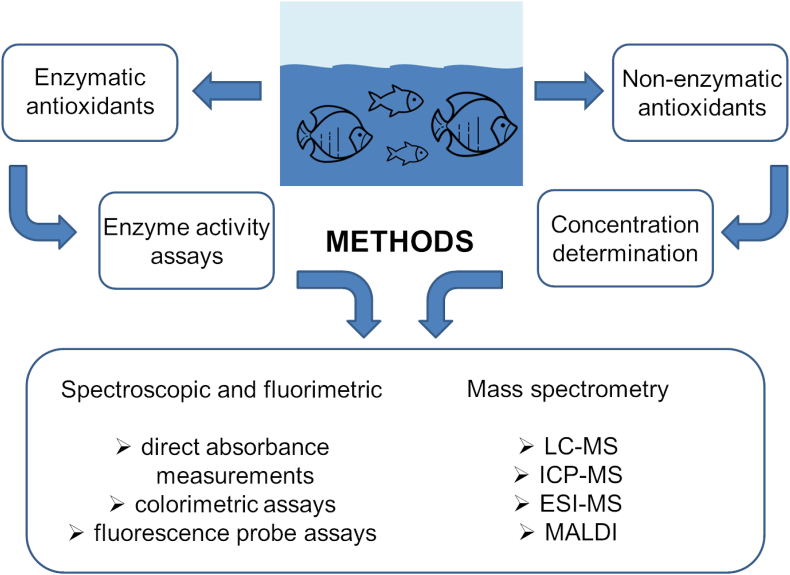
Schematic representation of methods used for oxidative stress biomarkers determination.

As mentioned in previous chapters, the toxicity of metallic NPs is related to the generation of ROS and constantly raising ROS production can trigger the induction of enzymatic and non-enzymatic antioxidants, which are commonly used as biomarkers. Assessment of these oxidative stress biomarkers provides useful and sensitive tools for monitoring the toxic effects of metallic NPs in aquatic organisms. Thus, the exact determination of their level offers an excellent opportunity to predict and analyze harmful effects induced by metallic NPs at a biochemical level. However, up to date, only several methods that enable the detection of biomarkers in very low concentrations have been developed (Fig. 7) (Vale et al., 2016; Katerji et al., 2019).

Throughout the literature, the most popular methods are based on spectrophotometry, fluorescence, and mass spectrometry measurements. This chapter summarizes and discusses methodology used for oxidative stress biomarkers determination in the aquatic environment. First, we critically describe each method's relevance and main challenges, followed by their thorough usage in the qualitative and quantitative determination of biomarkers levels.

The activity of enzymatic antioxidants, including superoxide SOD, CAT, GSTs, and GTx, have been widely quantified in many investigations (Farkas et al., 2011; Eroglu et al., 2015; Khan et al., 2017a; Lacave et al., 2017; Lu et al., 2017; Pecoraro et al., 2017; Abdelazim et al., 2018; Fkiri et al., 2018; Huang et al., 2018b; Saidani et al., 2019; Tang et al., 2019; Yang et al., 2022; Kakakhel et al., 2024). These methods investigate the level of the antioxidants before and after exposure to metallic NPs in the aquatic organisms. The concentration of these antioxidants depends on their formation and consumption during oxidative stress. Numerous spectrophotometric techniques have been designed to monitor enzymatic activity (Beauchamp and Fridovich, 1971; Misra and Fridovich, 1972; Habig et al., 1974; Marklund and Marklund, 1974; Lawrence and Burk, 1976; Oyanagui, 1984; Góth, 1991; Eyer et al., 2003; Katerji et al., 2019). Most of them are based on enzymatic reactions catalyzed by mentioned enzymes, with a specific substrate to observe changes in absorption within the UV–Vis range. Their total activity can be measured using a direct or indirect approach, for example, SOD activity can be measured directly based on the ability of SOD to inhibit the autoxidation of pyrogallol (Marklund and Marklund, 1974) or adrenaline (Misra and Fridovich, 1972) at λ = 420 nm or λ = 480 nm, respectively. Indirect determination of the SOD activity is based on its ability to scavenge superoxide radicals. The main principle of this approach is based on the reaction of nitro blue tetrazolium (NBT) reduction to blue formazan at λ = 560 nm, the SOD competes for the generated superoxide radicals and thus inhibits NBT reduction (Beauchamp and Fridovich, 1971; Oyanagui, 1984). Another direct quantitative spectrophotometric approach can be applied for measuring CAT activity, which is determined by the breakdown of H_2_O_2_ catalyzed by CAT and monitored by a decrease in absorbance of H_2_O_2_ solution at λ = 240 nm (Aebi, 1984). Alternatively, the CAT activity can be measured using another spectrophotometric assay based on measurements of the stable complex formation of H_2_O_2_ with ammonium molybdate at an absorbance of 405 nm (Góth, 1991). Similarly, GTx activity is measured by assessing the rate of hydrogen peroxide-induced oxidation of reduced GSH into GSSG. Because GSH forms a yellow product (TNB-) with 5,5′-dithiobisnitrobenzoic acid (DTNB), which absorbs light at λ = 412 nm, the GTx activity can be estimated as the amount that reduces the level of GSH (Lawrence and Burk, 1976; Eyer et al., 2003). Another important enzymatic biomarkers are GSTs, which play a significant physiological role in initiating the detoxication of potential alkylating agents; therefore, catalyze the reaction of thiol-containing compounds and, consequently, neutralize their electrophilic sites (Habig et al., 1974; Marklund and Marklund, 1974). In resemblance to mentioned techniques, the activity of GSTs is also based on a colorimetric approach; however, in this case, the ability of GST to conjugate 1-chloro-2,4-dinitrobenzene (CDNB) to GSH is accompanied by an increase in absorption at λ = 340 nm (Habig et al., 1974; Huang et al., 2018b). Overall, all the mentioned spectrophotometric methods are commonly used to measure enzymatic biomarkers levels in aquatic organisms because they are fast, simple, well understood for most applications, and can be recorded at a fixed time and kinetic mode (Table 2). In addition, assays based on colorimetric methods can be extended to efficient and highly sensitive immunological methods using microtiter plate-based ELISAs in combination with an automated spectrophotometer. Moreover, the spectrophotometric methods allow measurements of the full UV–Vis spectrum range, thus revealing possible sample contamination. Nevertheless, the dynamic range of detection, as well as sample conditions, limit the usage of spectrophotometric methods in biomarkers detection (Table 2). Even though the physical basis of spectrophotometric measurements is known, and the source of uncertainty can originate from instruments itself, less attention is paid to errors originate from the object under study and condition used. These include the concentration of biomarkers, temperature, pH, and impurities that may influence absorbance readout. All of these may result in incorrect absorbance reading, which can lead to over- or underestimation of biomarker level. However, the estimation of absorbance read out accuracy is not easy to determine. Sometimes it is possible to overreach the problem by correcting results using the procedural blank. Finally, selectivity is another issue, spectrophotometric methods do not allow discrimination between a biomarker of interest and contaminants or biomarkers isoforms that absorb at the same wavelength. Therefore, other methods should be used to offer higher specificity and accuracy (Table 2).

**Table 2:** Advantages and disadvantages of methods used for biomarkers determination. Consideration required for proper method selection.

METHODS	PROS	CONS	APPLICATION
Spectroscopy	➢ economic value – low cost ➢ easy and quick ➢ noninvasive ➢ measurement can be recorded at fixed time or kinetic mode	➢ low sensitivity ➢ sensitive to pH, temperature, and detergents ➢ poor selectivity ➢ stray light. Broadband detectors respond to all the light that reaches them ➢ low dynamic range	➢ enzyme activity assay for oxidative stress biomarkers detection
Fluorescence	➢ easy and quick ➢ noninvasive ➢ high sensitivity ➢ better selectivity ➢ fluorescent probes can enter cell and directly bind to thiols	➢ higher equipment cost ➢ possible fluorescence quenching ➢ fluorescent probes can be sensitive to pH ➢ some impurities may perturb fluorescence readout ➢ some fluorescent probes that enter the cell can be toxic at specific concentration	➢ determination of non-enzymatic antioxidants containing thiol groups
Mass spectrometry	➢ enable qualitative and quantitative analysis of metal ions (ICP-MS) ➢ quick response ➢ high selectivity and specificity ➢ large dynamic range ➢ high mass resolution ➢ high sensitivity ➢ coupled with HPLC – molecular sensitivity ➢ Covalent and non-covalent bonds destroyed (ICP-MS)	➢ highly expensive equipment as well as software (possibility of using free software)	➢ investigation of metalloproteins ➢ ESI-MS can be used for MT isoforms determination ➢ ICP-MS can be used for direct determination of metal content

In this case, fluorometric methods based on fluorescent probes like monochlorobimane (MCB) and monobromobimane (MBB) are frequently used (Farkas et al., 2011). They tend to form stable fluorescent adducts with non-enzymatic antioxidants like GSH and thiol-containing proteins, including MTs. Furthermore, a fluorogenic reagent 4-aminosulfonyl-7-fluoro-2,1,3-benzoxadiazole (ABD-F) for thiol labeling can be also used for determination of GSH and MTs (Krezel and Maret, 2016). Another probe which is worth mentioning is 6-iodoacetamidofluorescein (6-IAF) which enables the determination of reduced and oxidized states of MT and thionein (apoMT) (Haase and Maret, 2004). In some cases, other assays based on immunohistochemical protocols are used. In these assays, fluorescently labeled secondary antibodies are probes used for detection (Pecoraro et al., 2017). All of these probes have the substantial advantage of penetrating cells allowing analysis by commonly known techniques, including time-resolved or anisotropy decay fluorimetry, flow cytometry, and fluorescence microscopy (Farkas et al., 2011; Pecoraro et al., 2017). Even though fluorometric techniques quickly give a statistically relevant fluorescence signal with the utmost sensitivity and accurate detection reading, it should be mentioned that static (collisional) or dynamic (complex formation) quenching processes may occur. These significantly result in a decrease in fluorescence intensity. Moreover, fluorescence signals can also be affected by pH and environmental pollution; some impurities can absorb light and interfere with fluorescent readout (Table 2). Nevertheless, fluorescence-based methods are less laborious and require a short incubation time, thus providing a good alternative to spectrophotometry.

Currently, trace biological elements like Zn^2+^, Cu^2+^, Cd^2+^, and Fe^2+^ become another reliable oxidative stress biomarkers due to their essential role as cofactors of the enzymatic antioxidants. Therefore, robust qualitative and quantitative analysis of metals in aquatic systems is very important. To date, there are some fluorescent probes including FluoZin-3 and ZnAF-2F that allow the study of metal ions in biological systems (Hirano et al., 2002; Pluth et al., 2011; Marszalek et al., 2016; Hao et al., 2018). Also, great progress has been made in expanding the number of genetically encoded fluorescence sensors that can be used for monitoring intracellular transition metals. Such sensors enable the non-invasive fluorometric detection of intracellular transition metals and hence allow one to better understand the role of transition metal in both physiological and pathological conditions (Hao et al., 2018). Next to fluorometric method, another accurate and sensitive methods such as ultra-fast and high-performance liquid chromatography coupled with mass spectrometry (LC-MS) and especially inductively coupled plasma-mass spectrometry (ICP-MS) are commonly used techniques to detect metal ions in biological systems (Wind and Lehmann, 2004; Lobinski et al., 2006). Using plasma instead of other ionization sources prevents oxide formation and gives lots of other benefits including more efficient ionization (a trace amount of sample is needed) and a chemically inert environment (Table 2). Furthermore, this method opens doors for quantitative measurement of the total amount of the element of interest. Many limitations mentioned before can be overcome using ICP-MS, including an extensive dynamic range, versatility, and high sensitivity. Moreover, it should be emphasized that ICP-MS can be coupled with chromatography and electrophoresis, thus providing molecular specificity and becoming an attractive partner of electrospray and MALDI MS for the investigation of MTs as other important biomarkers for pollution in the aquatic organisms (Hauser-Davis et al., 2012; Gouveia et al., 2019). The ICP-MS gives more insightful data for biochemical application than fluorometric or spectrophotometry methods. Considering the countless possibilities that ICP-MS can offer for ecotoxicological study, its application in MTs study and MS data processing will be discussed later in the next chapter.

In conclusion, none of those methods and biomarkers are sufficient to discover the potentially toxic role of metallic NPs in the aquatic environment. There are no ideal biomarkers and techniques to analyze them, but some are more reliable than others. Nevertheless, no single parameter has yet been recommended to define the toxicological status of NPs, so yet no adequate comparison between biomarkers and methodologies used to measure them has yet been performed. Thus, it is difficult to make a reliable comparison between the toxic effect of NPs throughout different aquatic organisms. Only careful and critical evaluation of available methodologies and the use of several techniques enable researchers to assess ecotoxicological study properly.

## 5 Hyphenated mass spectrometry techniques for metal and thiol determination (ICP-MS, HPLC-ICP-MS, CE-ICP-MS, ESI-MS)

Mass spectrometry (MS) has become an indispensable tool for studying the metallome in cells. In the post-genomic era, it is essential to consider the roles of metal ions such as Cu^2+^/^+^, Fe^2+^/^3+^, or Zn^2+^ in understanding the various functions within cells. The concentration of metal ions plays a regulatory role in the function of many proteins. For example, Zn^2+^ is required for the proper folding of the transcription factor p53 (Ha et al., 2022). Conversely, several protein families are responsible for regulating metal ion levels in cells, such as ferritins for Fe and ceruloplasmin for Cu. One specific transport protein, MT, is unique in its ability to bind a wide range of metals. It plays a vital role in transporting and buffering essential metal ions such as Zn^2+^ or Cu^+^ to other proteins, as well as in detoxifying toxic metal ions such as Cd^2+^ or Hg^2+^ (Krezel and Maret, 2021). Another important aspect to consider is how the chemical form of an element determines its biological or toxicological properties. For example, soluble Ni^2+^ salts like NiCl_2_ or NiSO_4_ are less toxic compared to Ni_3_S_2_, which is a potent carcinogen (Fletcher et al., 1994). Therefore, understanding the distribution of metals among different proteins, their localization, and their chemical speciation is crucial for comprehending the toxicological properties of metal elements.

In recent years, the field of metallomics has emerged due to advancements in instrumentation. Analyzing biological samples, which are complex and contain low levels of metal species, requires techniques with high resolution for protein separation and high sensitivity and selectivity for metal determination. Therefore, the analytical techniques used should provide information about: (i) the localization of metal elements in cells, including determining their distribution among cellular compartments; (ii) the ligand donors to which metals are coordinated; and (iii) the concentration of specific metal species. To achieve this, standalone or hyphenated MS techniques are employed. In this section, we will briefly introduce the most commonly used analytical methods and provide examples of their applications, focusing on the determination of MT proteins and metal ions in the aquatic environment (Table 3).

**Table 3:** The review of methods used for MT and metal detection in aquatic model organisms.

Organism	Analyte	Method	Ref
*P. argentinus*	MT	DPP	(Bertrand et al., 2016)
Zebrafish embryos	MT	RT-qPCR	(Brun et al., 2014)
Zebrafish embryos	Zn	ICP-MS	(Brun et al., 2014)
Fish species	MT	DPP	(Siscar et al., 2014)
Fish species	Ag, Cd, Cr, Cu, Fe, Hg, Pb, Se and Zn	ICP-MS	(Siscar et al., 2014)
*C. auratus gibelio*	Cd, Cu and Zn	ICP-MS	(Van Campenhout et al., 2010)
*C. auratus gibelio*	Metal-MT	SEC-ICP-MS	(Van Campenhout et al., 2010)
*S. tiburo*	Hg	AAS	(Walker et al., 2014)
*S. tiburo*	MT	Immunoblotting	(Walker et al., 2014)
Mussel	Cd, Cu	ICP-MS	(Lavilla et al., 2012)
Fish species	Cd, Zn, Pb, Cu and Hg	SEC-ICP-MS	(Hauser-Davis et al., 2012)
Snails *M. cornuarietis* and *P. bridgesi*	Cd, Cu, Zn	SEC-ICP-MS	(Maltez et al., 2009)
Snails *M. cornuarietis* and *P. bridgesi*	MT	MALDI-TOF	(Maltez et al., 2009)
Mussels, *M. galloprovincialis*	Al, Ba, Cu, Fe, Mn, Sr, Zn, MT-like	HPLC-ICP-OES	(Santiago-Rivas et al., 2007)
Gibel carp, *C. auratus gibelio*	Cu, Zn, Cd and MT	HPLC-ICP-MS	(Infante et al., 2006)
Fish species: *G. brasiliensis* and *N. barba*.	Cu, Zn, Cd and MT	HPLC-ICP-MS	(Rodriguez-Cea et al., 2006)
Larvae *C. gigas*	Cu, Cd	AAS	(Damiens et al., 2006)
Larvae *C. gigas*	MT	DPP	(Damiens et al., 2006)
Snails *P. bridgesii* and *B. glabrata*	Cu, Zn, Cd and MT	ESI-MS	(Dallinger et al., 2020)
Marine snail L. *littorea*	Cu, Zn, Cd and MT	ESI-MS	(Baumann et al., 2017)
Mollusc L. *gigantea*	Cu, Zn, Cd and MT	ESI-MS	(Calatayud et al., 2021)

DPP: differential pulse polarography. AAS: atomic absorption spectrophotometry.

### 5.1 Standalone MS techniques

Regardless of the mass spectrometer configuration, the three main ionization sources are inductively coupled plasma (ICP), electrospray ionization (ESI), and matrix-assisted laser desorption/ionization (MALDI). Other ionization sources, such as atmospheric pressure chemical ionization (APCI), electron ionization (EI), desorption ESI (DESI), or laser-induced liquid bead ion desorption (LILBID), should also be mentioned (Cernescu et al., 2012; Peetz et al., 2019). It is important to note that no single ionization technique can fully characterize all components present in a biological sample (Bhardwaj and Hanley, 2014). The choice of ionization technique depends on factors such as the sample's polarity, size, and the specific analysis of interest. Although there is overlap in the range of applicability among some ionization sources, certain techniques are better suited for specific sample species, especially when coupled to a separation system. Therefore, the selection of the ionization technique depends on the availability of the instrumentation. The second part of the mass spectrometer is the mass analyzer. The ICP ionization source is typically coupled with quadrupole and time-of-flight (TOF) mass analyzers, while MALDI is coupled with TOF. ESI is compatible with most available mass analyzers, including quadrupole, TOF, and Orbitrap. In this text, we will use the general term “MS” without specifying the particular mass analyzer.

**Fig. 8:**
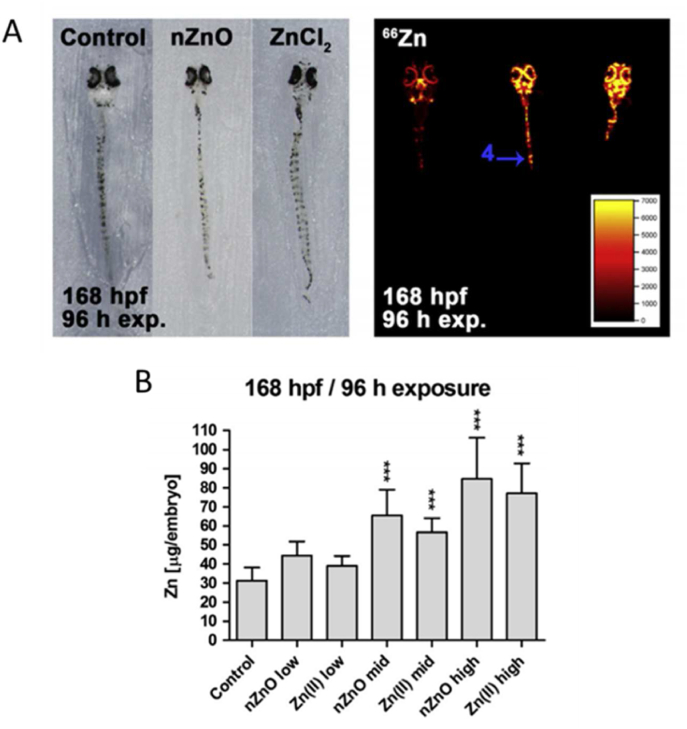
Study of the effect of zinc oxide nanoparticles (ZnO NPs) and Zn^2+^ ions on zebrafish embryos (Brun et al., 2014). A) Laser ablation-ICP-MS shows that the uptake and tissue distribution for Zn^2+^ were identical for ZnO NPs and for Zn^2+^ (dissolved ZnCl_2_) in eleuthero-embryos. B) Determination of the total Zn content by ICP-MS shows identical results for ZnO NPs and for Zn^2+^ (dissolved ZnCl_2_) in eleuthero-embryos (adopted from (Brun et al., 2014)).

ICP-MS is one of the most commonly used techniques for metal analysis, offering remarkable sensitivity (sub-ppt) for determining the composition of metal elements. However, in its standalone form, ICP-MS only provides information about the total content of a particular metal element and does not reveal metal speciation. To determine metal speciation, ICP-MS is typically coupled with a separation technique such as HPLC or CE. ICP is a multi-element ion source, allowing simultaneous measurement of several elements. An interesting application is the quantification of an element's concentration. When the molecule concentration is low enough, matrix interference from the molecule's surroundings can be considered negligible. In such cases, inorganic elemental standards can be used for quantification, achieving an accuracy of 10% or better (Svantesson et al., 2002). For instance, Brun et al. studied the effects of ZnO NPs and Zn^2+^ ions on zebrafish embryos. Using ICP-MS and laser ablation-ICP-MS, the authors determined that the total Zn^2+^ content, uptake, and tissue distribution were identical for both ZnO NPs and dissolved Zn^2+^ (ZnCl_2_) in eleuthero-embryos. Specifically, Zn^2+^ ions were found in the retina/pigment layer of the eyes and in the brain, rather than being merely adsorbed on the skin surface (Fig. 8).

Both ZnO NPs and Zn^2+^ ions induced inhibition effects, as indicated by similar transcriptional expression measured by RT-qPCR. Brun et al. detected the induction of MT-2 upon incubation with ZnO NPs or Zn^2+^ and concluded that the primary source of the observed effects is the dissociation of Zn^2+^ from ZnO NPs in water (Brun et al., 2014). Siscar et al. used ICP-MS to determine the concentration of nine metal ions in the liver of seven deep-sea fish species. Their analysis revealed that the metal levels in these Mediterranean deep-sea fish species were intermediate compared to equivalent fish species from Atlantic waters or hydrothermal vents. They also found that MT, Se^2+^, and Zn^2+^ played a role in metal protection (Siscar et al., 2014). In other studies, the cytosolic metal concentrations of Cd^2+^, Cu^+^/^2+^, and Zn^2+^ were determined in the gills, liver, and kidneys of gibel carp to assess the impact of environmental metal contamination (Van Campenhout et al., 2010).

While ICP-MS is a superior technique in terms of detection limits and multi-elemental analysis, inexpensive atomic absorption spectroscopy (AAS) is still used for analyzing specific metal elements. For example, Damients et al. measured increased MT levels upon Cd^2+^ and Cu^+^/^2+^ exposure for 24 h in larvae of *C. gigas* using differential pulse polarography (DPP). The metal bioaccumulation determined by AAS indicated that MT might function as a detoxification mechanism and could be used as a biomarker for measuring metal accumulation (Damiens et al., 2006). Interestingly, Walker et al. did not find a relationship between muscle Hg^2+^ concentrations (total concentration determined by AAS) and muscle/hepatic levels of MT measured by immunoblotting in *S. tiburo* (Walker et al., 2014).

**Fig. 9:**
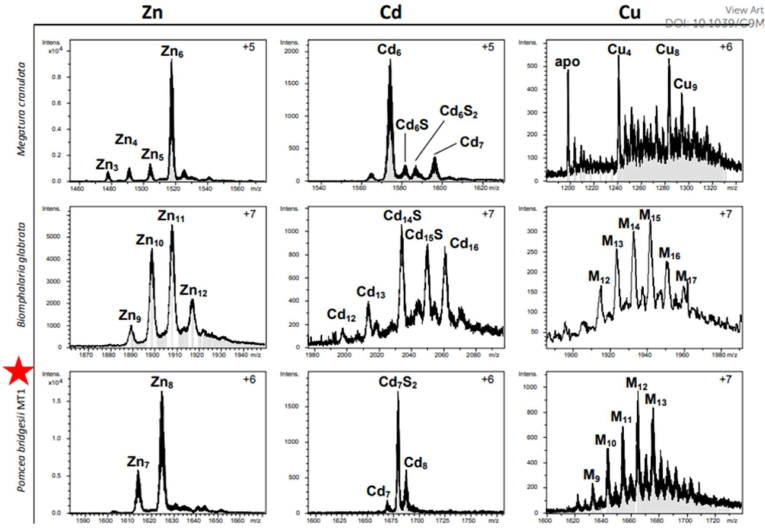
ESI-MS spectra of Pomacea bridgesii, *Biomphalaria glabrata* and *Megathura crenulata* obtained under nondenaturing conditions (Dallinger et al., 2020). The mass spectra of Pomacea bridgesii and *Biomphalaria glabrata* resembles to *Megathura crenulata*. Because the latter possesses an unspecific MT that produces a mixture of sulfide-containing heterometallic complexes in Cd^2+^-enriched media, the former species are non-selective to Cd^2+^ ions (adopted from (Dallinger et al., 2020)).

ESI-MS is particularly relevant for studying metal-binding proteins. During the 1990s, it was demonstrated that ESI-MS could be used for the analysis of protein complexes involving noncovalent interactions (Ganem et al., 1991; Loo et al., 1993; Robinson et al., 1994; Rostom and Robinson, 1999). In a recent work by Dallinger et al., a comprehensive metallomics strategy was employed to demonstrate how Cd^2+^ drove the convergent evolution of Cd^2+^-selective MT gastropod clades. The authors used ESI-MS to decipher the metal-protein stoichiometry and defined metal selectivity based on the formation of homometallic complexes with cognate metal ions and the formation of heterometallic mixtures with non-cognate metal ions. The mass spectra from *Pomacea bridgesii* and *Biomphalaria glabrata* resembled those of *Megathura crenulata*, which possesses an unspecific MT that produces a mixture of sulfide-containing heterometallic complexes in Cd^2+^-enriched media (Fig. 9).

Therefore, the authors concluded, using ESI-MS among other techniques, that the Cd^2+^ selectivity in MTs has been lost in snails that adapted to freshwater habitats since the Cretaceous period (Dallinger et al., 2020). Baumann et al. determined the NMR structure of the marine snail *Littorina littorea* in complex with Cd^2+^. Analysis by ESI-MS showed that the protein bound up to 9 Zn^2+^ or Cd^2+^ ions, coordinated in three domains. The formation of a three-domain MT can be seen as a structural variation resulting from evolution. Some gastropod lineages have changed their habitat and had to adapt to new environments with different metal ion availabilities. The structural evolution favored these species in coping with Cd^2+^ stress and bioaccumulation (Baumann et al., 2017). In another example, the metal binding preferences of the recombinant MT protein from the mollusk *L. gigantea* were studied using ESI-MS and ICP-AES. The evolution of MT seemed to result in the formation of new structural domains that increased metal binding capacity. *L. gigantea* did not show any metal preference between Zn^2+^ or Cu^2+^ ions. However, with Cd-supplemented media, only Cd_7_-Lgi species were observed, indicating the formation of a more thermodynamically favored Cd-cluster (Calatayud et al., 2021).

The use of MALDI-MS in the analysis of MT in aquatic environments has been limited due to the difficulty in preserving metal-protein complexes during MALDI ionization. Therefore, MALDI-MS has mainly been used for measuring the molecular mass of polypeptides or the fragments derived from enzymatic digestion of proteins (Maltez et al., 2009).

### 5.2 Hyphenated MS techniques

In hyphenated MS techniques, the molecular separation of complex samples containing mixtures of proteins, cells, or cellular compartments prior to detection becomes important. While ESI-MS can sometimes deconvolute a mixture of proteins without separation, the physicochemical separation of a mixture simplifies the analysis of mass spectra. In the case of ICP-MS, speciation is lost without a separation technique. Several separation techniques can be coupled online or offline to mass spectrometry detection. Bertrand et al. studied the effect of the organophosphorus pesticide chlorpyrifos (CPF) in the environment. They used gas chromatography coupled with an electron capture detector (GC-ECD) for the determination of volatile compounds and an LC-MS (HPLC-ESI-qTOF) system for non-volatile compounds (Bertrand et al., 2016). Although GC has limited applicability in bioinorganic speciation, it can be useful for analyzing volatile sulfur and selenium species (Szpunar, 2000). Van Campenhout et al. determined the metal content in different cytosolic fractions from gibel carp using an LC-ICP-MS system. Size-exclusion chromatography (SEC) was selected as the separation mechanism, but the authors found higher metal values in the total tissue analysis by ICP-MS, suggesting that some sample fraction was not eluted from the SEC column and therefore not analyzed by ICP-MS (Van Campenhout et al., 2010). Infante et al. used anion exchange as the separation mechanism for HPLC-ICP-MS to study the metal speciation of MT isoforms in gibel carp exposed to environmental metal pollution. They fractionated up to five MT isoforms with online measurement of metal content using ICP-MS (Infante et al., 2006).

Rodríguez-Cea et al. performed metal speciation analysis of MTs in two tropical fish species, *G. brasiliensis* and *N. barba*. They used SEC to separate cytosolic proteins and anion-exchange chromatography coupled with ICP-MS to analyze the MT fraction. The MT isoforms in these species differed from other fish liver MTs, and the presence of Cd^2+^ in a specific MT isoform may be related to its role in detoxifying metal ions (Rodriguez-Cea et al., 2006). MT in fish bile was also analyzed using SEC-ICP-MS, revealing distinct peaks for Hg^2+^, Zn^2+^, and Cu^+^/^2+^ and indicating Hg^2+^ contamination in Jacarepaguá Lagoon (Hauser-Davis et al., 2012).

Santiago-Rivas et al. developed a method for determining metal-binding MT-like proteins in mussels (*M. galloprovincialis*) using cytosolic preparation and analysis by anion exchange HPLC-ICP-OES (Santiago-Rivas et al., 2007). Lavilla et al. improved this method by simplifying the experimental setup and replacing OES with MS detection (Lavilla et al., 2012). Finally, MT-like proteins in two snail species, *M. cornuarietis* and *P. Bridgesi*, were analyzed for metal speciation using SEC-ICP-MS and anion-exchange-FPLC hyphenated to ICP-MS. MALDI-TOF and peptide-mass fingerprinting were also employed for MT isoform identification. The authors reported higher Cd^2+^ accumulation in *M. cornuarietis*, making it a good candidate for monitoring metal contamination in freshwater ecosystems (Maltez et al., 2009).

In conclusion, the examples presented in this section demonstrate the applications of standalone and hyphenated MS techniques in the analysis of MTs and metal ions. These techniques have provided insights into important issues such as metal contamination, structural evolution, and metal speciation in different tissues.

## 6 Conclusions and outlooks

Currently, NPs are widely used in many industries, making it crucial to understand their fate, behavior, and potential effects on organisms in the aquatic environment. The fate of NPs is highly dependent on their physicochemical properties and the surrounding environment. However, despite numerous studies, there is still insufficient information to establish a clear relationship between the properties of NPs, the environment, and their toxicity to aquatic organisms. Inconsistencies and conflicting information arise from differences in experimental design, composition of exposure media, model organisms, and inadequate characterization of nanomaterials. Establishing standardized protocols for nanoparticle characterization and toxicity testing would ensure consistency and reproducibility of results. But standard procedures for studying NPs' toxicity are yet to be established. Nevertheless, biomarkers of oxidative stress became essential tools in aquatic toxicology, providing critical information on the physiological responses of organisms to environmental stressors. Many studies have evaluated the toxicity of metallic NPs on aquatic organisms based on antioxidant-related enzymes and peptides, metallothionein, or other biomarkers of oxidative stress. Measuring these biomarkers in aquatic organisms, such as fish, bivalves, algae, and invertebrates, helps to provide valuable insights into the overall health of aquatic ecosystems and assessing the impact of NPs. Furthermore, monitoring these biomarkers in response to metallic NPs exposure can not only enhance our understanding of the nanoparticle mechanisms of toxicity, but also can contribute to the development of strategies for ecological risk assessments to mitigate the adverse effects of NPs in the aquatic environment. Alterations in antioxidant enzyme activities and oxidative damage levels may indicate the presence of pollutants in the water, helping to identify areas at risk and prompting swift intervention to mitigate potential harm to aquatic life. Monitoring oxidative stress can be potentially used for tracking and evaluating the effectiveness of remediation strategies, such as water treatment processes or habitat restoration. Furthermore, knowledge of the ecotoxicity of NPs can also contribute to the development of safer nanomaterials. Currently, various techniques are available to assess oxidative stress, but careful selection of suitable methods is essential due to limitations associated with each technique. Additionally, there is no ideal biological model for adequately assessing the long-term toxic effects induced by metallic NPs in aquatic systems. Although, comparative studies across different aquatic species can provide valuable insights into species-specific responses to oxidative stress induced by metallic NPs, understanding interspecies variations in biomarker responses and establish a clear conclusion of toxicity of NPs is very demanding. Therefore, comparing biomarkers and methods remains a significant challenge in determining NPs' ecotoxicity. To obtain high-quality toxicological data, it is necessary to use multiple techniques after critically evaluating available methodologies. Future studies can explore the development and implementation of advanced analytical techniques for detecting and quantifying oxidative stress biomarkers in aquatic organisms with higher sensitivity and specificity. This includes the integration of cutting-edge technologies such as mass spectrometry, fluorescence, and spectrophotometry to enhance the accuracy and efficiency of biomarker measurements. Promising direction for future research is also development of innovative analytical techniques for monitoring oxidative stress biomarkers in real-time. Furthermore, integrating multi-omics approaches with analysis of stress biomarkers can offer a comprehensive understanding of the molecular mechanisms underlying nanoparticle-induced toxicity in aquatic organisms. This holistic approach can reveal intricate biological responses and potential biomolecular pathways affected metallic NPs. Still evolving computational modeling and simulation can be also used for prediction of the toxicological outcomes of NPs, reducing the need for extensive animal testing. Use of *in silico* models to simulate nanoparticle interactions with biological systems can provide insights into potential toxicity and mechanisms of action. Moreover, there is a lack of the long-term and chronic exposure studies, as acute toxicity studies may not capture the full spectrum of potential adverse effects. Therefore, further studies regarding the long-term impacts of metallic NPs on aquatic ecosystems should be one of the aims of interest.

## CRediT authorship contribution statement

**Tomas Do:** Writing – original draft. **Silvia Vaculciakova:** Writing – original draft. **Katarzyna Kluska:** Writing – original draft. **Manuel David Peris-Díaz:** Writing – review & editing, Writing – original draft. **Jan Priborsky:** Writing – review & editing. **Roman Guran:** Writing – review & editing, Formal analysis. **Artur Krężel:** Writing – review & editing, Conceptualization. **Vojtech Adam:** Writing – review & editing, Funding acquisition. **Ondrej Zitka:** Writing – review & editing, Funding acquisition, Conceptualization.

## Declaration of competing interest

The authors declare that they have no known competing financial interests or personal relationships that could have appeared to influence the work reported in this paper.

## Data Availability

No data was used for the research described in the article.
